# Cellular Interrogation: Exploiting Cell-to-Cell Variability to Discriminate Regulatory Mechanisms in Oscillatory Signalling

**DOI:** 10.1371/journal.pcbi.1004995

**Published:** 2016-07-01

**Authors:** Javier Estrada, Natalie Andrew, Daniel Gibson, Frederick Chang, Florian Gnad, Jeremy Gunawardena

**Affiliations:** 1 Departamento de Física de la Materia Condensada and Instituto Nicolás Cabrera, Universidad Autónoma de Madrid, Madrid, Spain; 2 John A. Paulson School of Engineering and Applied Sciences, Harvard University, Cambridge, Massachusetts, United States of America; 3 Department of Systems Biology, Harvard Medical School, Boston, Massachusetts, United States of America; 4 Molecular and Cell Biology Graduate Program, Harvard University, Cambridge, Massachusetts, United States of America; University of Virginia, UNITED STATES

## Abstract

The molecular complexity within a cell may be seen as an evolutionary response to the external complexity of the cell’s environment. This suggests that the external environment may be harnessed to interrogate the cell’s internal molecular architecture. Cells, however, are not only nonlinear and non-stationary, but also exhibit heterogeneous responses within a clonal, isogenic population. In effect, each cell undertakes its own experiment. Here, we develop a method of cellular interrogation using programmable microfluidic devices which exploits the additional information present in cell-to-cell variation, without requiring model parameters to be fitted to data. We focussed on Ca^2+^ signalling in response to hormone stimulation, which exhibits oscillatory spiking in many cell types and chose eight models of Ca^2+^ signalling networks which exhibit similar behaviour in simulation. We developed a nonlinear frequency analysis for non-stationary responses, which could classify models into groups under parameter variation, but found that this question alone was unable to distinguish critical feedback loops. We further developed a nonlinear amplitude analysis and found that the combination of both questions ruled out six of the models as inconsistent with the experimentally-observed dynamics and heterogeneity. The two models that survived the double interrogation were mathematically different but schematically identical and yielded the same unexpected predictions that we confirmed experimentally. Further analysis showed that subtle mathematical details can markedly influence non-stationary responses under parameter variation, emphasising the difficulty of finding a “correct” model. By developing questions for the pathway being studied, and designing more versatile microfluidics, cellular interrogation holds promise as a systematic strategy that can complement direct intervention by genetics or pharmacology.

## Author Summary

We have developed a cellular interrogation methodology that combines programmable microfluidics, fluorescence microscopy and mathematical analysis and have used it to discriminate between models of repetitive Ca^2+^ spiking in HeLa cells. Our approach exploits the natural variability in response of individual cells in a clonal population and the non-steady state behavior of the response in each cell, thereby providing more powerful discrimination. Interrogation consists of steps or pulses of histamine of fixed concentration and width but varying frequency. Eight mathematical models of repetitive Ca^2+^ spiking were chosen from the literature and methods of nonlinear frequency and nonlinear amplitude analysis were developed which ruled out all but two of the models, without having to fit the models to the data. Further analysis of the remaining models yielded predictions that were experimentally confirmed. Cellular interrogation offers a general approach to ruling out competing hypotheses about molecular mechanisms, which is complementary to traditional methods of genetics and biochemistry.

## Introduction

The divalent calcium cation, Ca^2+^, occupies an unusual position in respect of cellular behaviour. It is highly toxic, being especially prone to precipitate phosphate, and most cells go to considerable lengths to exclude it, typically maintaining a 20,000-fold differential between the concentration of Ca^2+^ in the extracellular medium, in the low millimolar range, and the typical concentration in the cytoplasm, of around 100nM [1]. At the same time, Ca^2+^ is widely used as an intracellular second messenger and the substantial transmembrane potential difference makes it particularly useful for the fastest cellular events, such as synaptic release of neurotransmitters. In view of the tension between these features, it is not surprising that the dynamics of intracellular Ca^2+^ show distinctive patterns in time and space.

In response to stimulation by a variety of hormones and other agonists, mammalian cells exhibit repetitive spikes of cytoplasmic Ca^2+^ [2, 3], thereby allowing Ca^2+^ to be deployed without accumulating to toxic levels. The frequency of oscillations often increases with agonist concentration and downstream cellular processes which are sensitive to Ca^2+^ can show striking frequency dependence. This has suggested that Ca^2+^ implements a frequency-modulated form of information processing, allowing exogenous signals to selectively tune a broad range of cellular responses [4].

**Fig 1:**
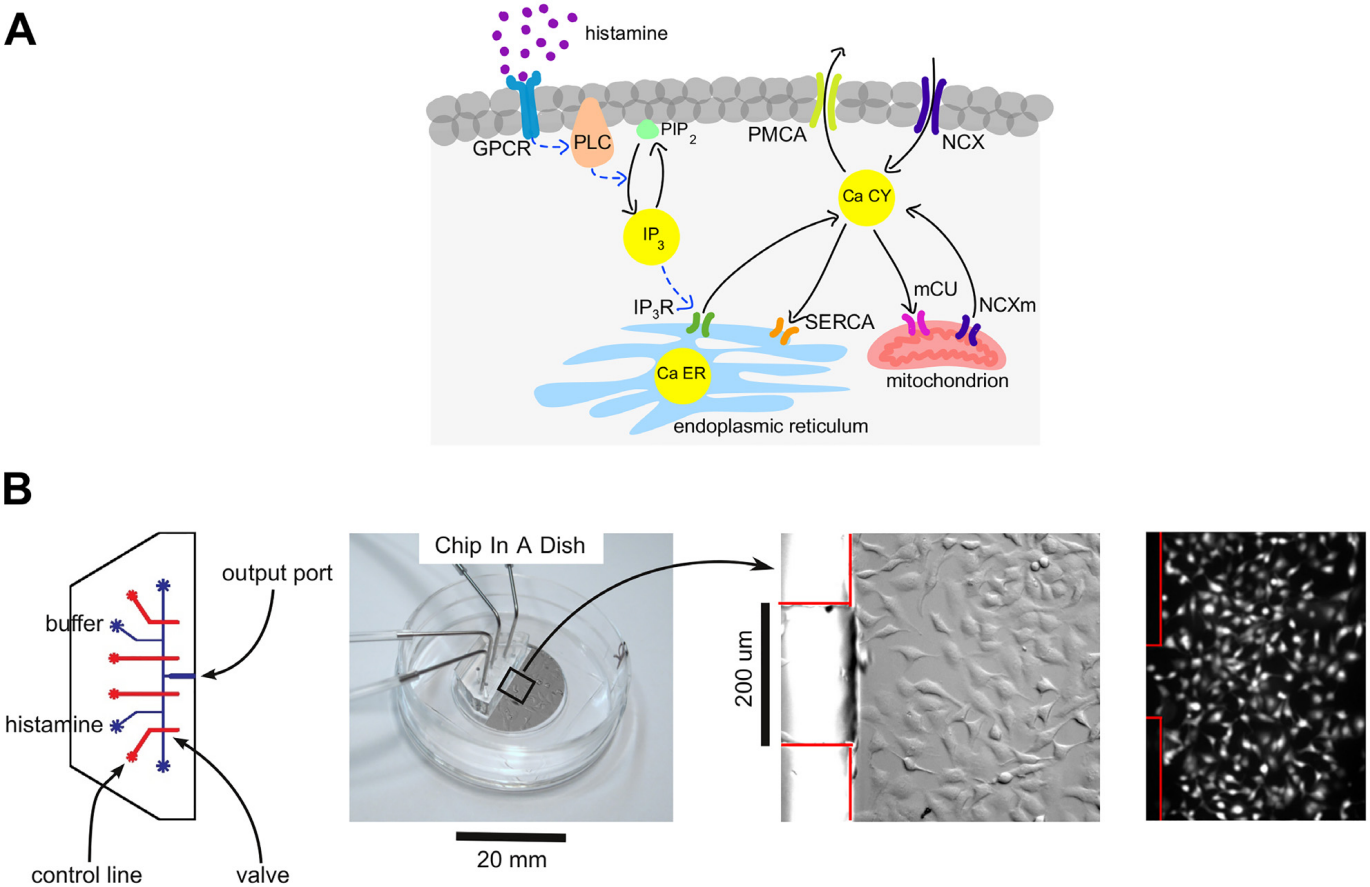
Cellular circuitry and experimental methodology. **(A)** Components of the calcium signalling toolkit are shown, as relevant to the models discussed in this paper. Yellow circles represent the principal small-molecule species involved in calcium dynamics, IP_3_, Ca^2+^ in the cytoplasm (Ca CY) and Ca^2+^ in the ER (Ca ER). Black lines denote Ca^2+^ fluxes, dashed blue lines denote activation. The positive and negative feedbacks between the components are illustrated in Fig 3. Abbreviations used: GPCR, G-protein coupled receptor; IP_3_, inositol-1,4,5-trisphosphate; IP_3_ R, IP_3_ receptor; NCX, Na^+^-Ca^2+^ exchanger; PIP_2_, phosphatidylinositol-4,5-bisphosphate; PLC, phospholipase C; PMCA, plasma membrane Ca^2+^ -ATPase; SERCA, sarco/endo-plasmic reticulum Ca^2+^ -ATPase; mCU, mitochondrial Ca^2+^ uniporter; NCXm, mitochondrial Na^+^-Ca^2+^ exchanger. **(B)** Microfluidic platform. From left to right: first, a schematic of the two-layer PDMS device. Buffer and histamine plus buffer are provided through the indicated flow lines (blue) at 5 psig. Valves are regulated by computer through the control lines (red), operating at 25 psig, to supply histamine or buffer to the output port, thereby generating steps or pulses, as required. Second, a photograph of the device bonded to the glass-bottomed dish (Chip-In-A-Dish), showing the four tubes leading to the control lines. Third, a differential interference contrast image of HeLa cells growing in the dish next to the output port, with the device border outlined in red. Fourth, fluorescence microscopy image, showing typical Fluo4 fluorescence in response to histamine stimulation.

To orchestrate the information processing required for a given cell type and physiological context, cells exploit a conserved calcium signalling “molecular toolkit” [5], from which appropriate Ca^2+^ -handling proteins are picked and mixed (Fig 1A). Several molecular networks have been put forward to account for the Ca^2+^ oscillations, typically based on interlinked positive and negative feedback loops, a motif that is believed to implement robust frequency control [6]. The corresponding mathematical models show stable limit-cycle oscillations which resemble those shown by Ca^2+^, as reviewed in [7, 8]. However, it has proved difficult to conclusively determine which network is responsible for Ca^2+^ oscillations in any cell type.

We approached this problem by asking whether cells could be probed with more complex forms of stimulation in such a way that their responses told us more about the underlying network. It is well known, for instance, that a linear system can be reconstructed from its response to different frequencies of stimulation (such engineering analogies are reviewed further in the Discussion) and this strategy has been explored previously in other signalling networks [9–12].

There are several difficulties in applying this strategy to Ca^2+^ signalling. In addition to being nonlinear, Ca^2+^ responses are also non-stationary, with neither the amplitude nor the period of spikes reaching a steady state, and different cells, even in a clonal population, can exhibit markedly different oscillatory responses [13], making it difficult to glean information from population-averaged measurements. However, the problem of cell-to-cell variation may potentially be turned to our advantage. It seems reasonable to assume that each cell in a clonal, isogenic population has the same network and what makes the cells respond differently is that the effective parameters of the network differ between cells. Accordingly, by examining single-cell responses and comparing the experimental responses to that of a mathematical model exposed to parameter variation, it might, in principle, be possible to gain more information with which to constrain the underlying network. Cell-to-cell variation has also been exploited in other ways [14, 15] and its potential as a methodology has been noted [16].

To develop our strategy, we designed and built two-layer polydimethylsiloxane (PDMS) microfluidic devices which can reproducibly generate a train of pulses of an appropriate hormone, such as histamine (Fig 1B and Materials and Methods). Pressure-regulated on-chip valves [17] allow pulse width and inter-pulse period to be controlled by computer. To avoid cellular stress responses due to growth on PDMS and exposure to high shear flow, the device was bonded to a dish in which the cells were grown as normal (“Chip-In-A-Dish”). Total cellular Ca^2+^ was measured at single-cell resolution using the cell-permeant Ca^2+^ -sensitive dye Fluo4-AM and fluorescence microscopy.

**Fig 2:**
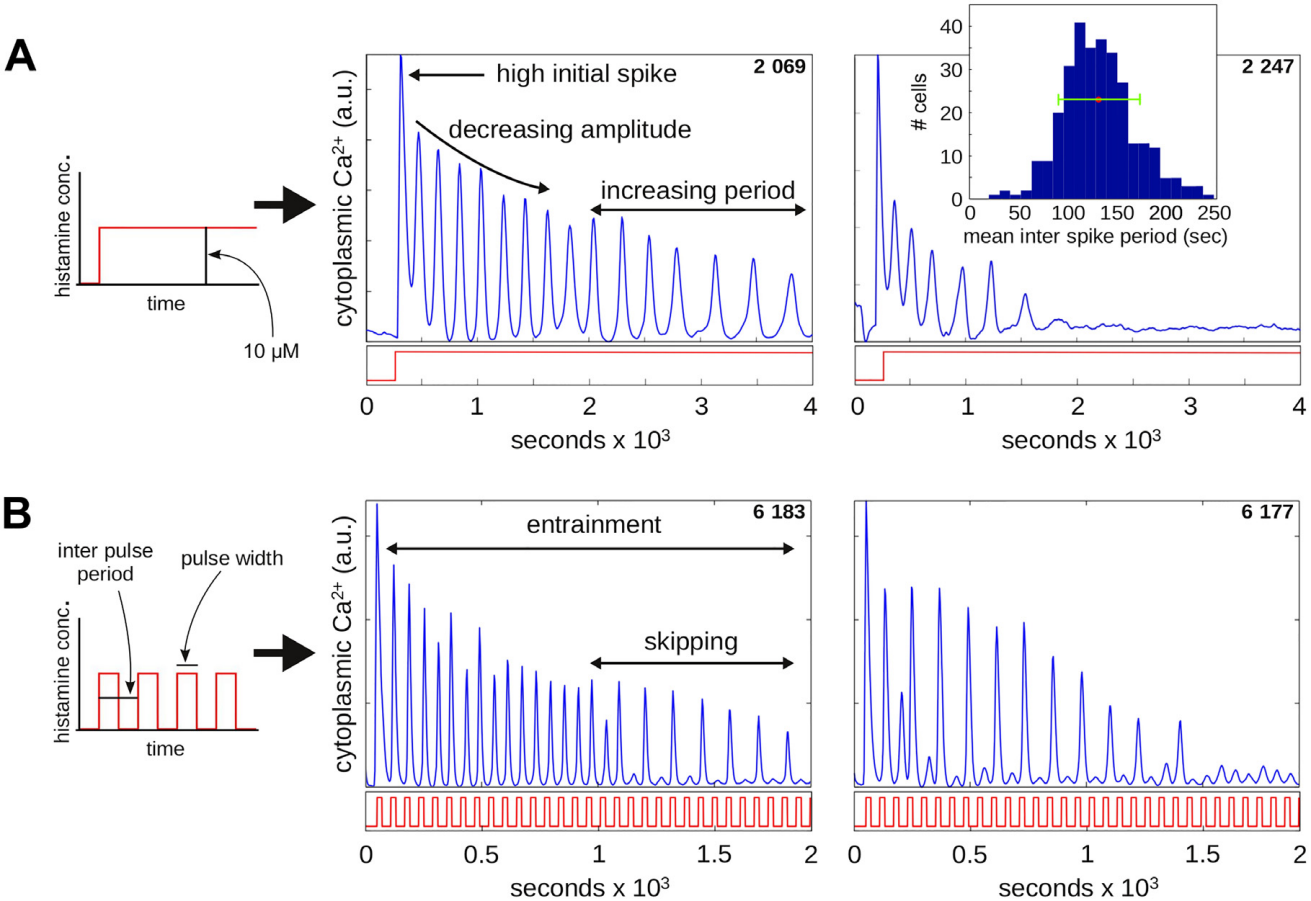
Cellular responses to steps and pulses. **(A)** Plots of two cells (identified in the top right-hand corner by experiment number and cell number as listed in S1 Text), showing effective Ca^2+^ concentration in the cytoplasm as a function of time, in response to a step of 10 *μ*M histamine, illustrated in the graph on the left and also beneath each plot in red. The inset shows a histogram of mean inter-spike periods over all cells for three step experiments (experiment numbers 1–3 in S1 Text), with the green bar showing a mean ± SD of 130 ± 40. **(B)** Plots of two cells, as previously, in response to repetitive pulses of 10 *μ*M histamine with a pulse width of 22 seconds and an inter-pulse period of 60 seconds, as defined in the graph on the left.

Fig 2A and 2B show features of the Ca^2+^ response of individual cells to steps and pulses, respectively, of histamine. We kept to a fixed amplitude of 10 *μ*M histamine throughout. A step increase elicits a large initial Ca^2+^ spike followed by repeated spikes with increasing inter-spike periods. The time-averaged period has a roughly Gaussian distribution over the cell population, with a mean ± SD of 130 ± 40 seconds (inset). This “natural period” gives a timescale for the free-running oscillator.

Repetitive histamine pulsing at a smaller-than-natural period entrains the oscillator. Initially, each histamine pulse elicits a Ca^2+^ spike but this leads eventually to skipping, with some pulses generating only tiny spikes, followed again by larger ones (Fig 1C). Such “phase locking” is well known in forced oscillators [18]. It is visible in early calcium-spiking data [19] and has been studied previously [20, 21]. In our experiments, the relative timescale of pulse to step stimulation, given by the ratio of pulse width in seconds to the mean natural period of 130 seconds, varied between 5/130 (0.04) and 38/130 (0.29). Note that both step and pulse stimulations elicit non-stationary responses.

**Fig 3:**
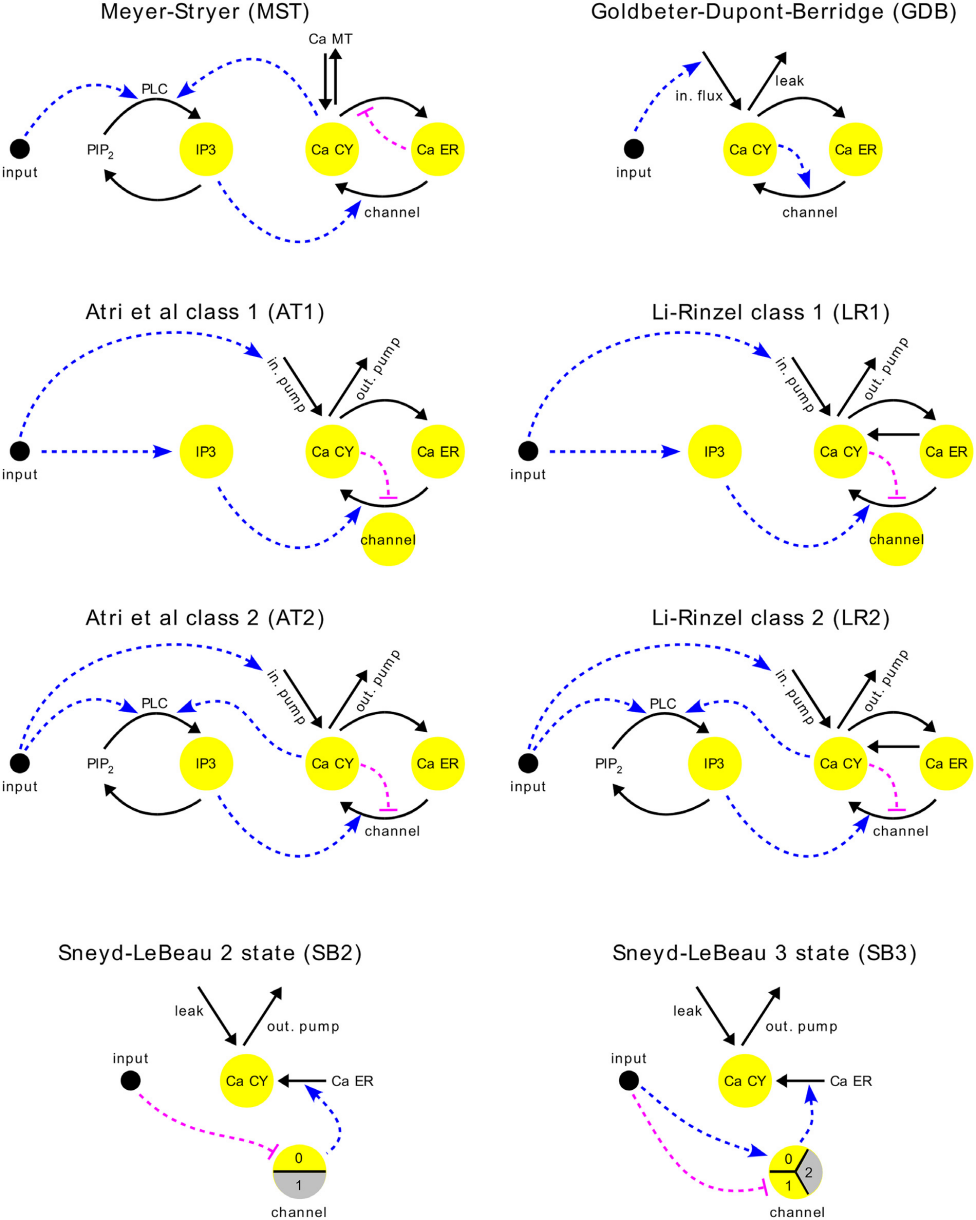
Models of oscillatory Ca^2+^ spiking. Eight schematic molecular networks are shown; citations to the original papers are given in S1 Text along with detailed mathematical descriptions. Yellow discs or disc-segments show the dynamical variables in each model. Thick black arrows show fluxes of Ca^2+^ between compartments or fluxes between phospho-inositol moieties; arrows with no source or no target show Ca^2+^ fluxes from or to, respectively, the extra-cellular compartment. Dashed lines show positive (blue, arrow) and negative (magenta bar) influences; additional positive or negative influences may arise through the details of the mechanism behind each individual flux. Abbreviations are as in Fig 1A.

To relate this data to the circuitry within cells, we examined eight networks, for which mathematical models exhibit oscillatory spiking (Fig 3). We chose these models to include both small distinctions (SB2 vs SB3) and large (MST vs GDB), as well as the important distinction [22] between class 1, or receptor-controlled, (AT1, LR1), and class 2, or second-messenger controlled, (AT2, LR2, MST), models, which is discussed further below. In separate work, we developed a computational infrastructure called Proteus for building a spectrum of models in a modular fashion from a basic set of components [23], in keeping with the idea of a calcium signalling toolkit. All the models used in this paper are publicly accessible through the Proteus website.

The chosen models exhibit oscillatory spiking in response to step stimulation, eventually reaching a stable limit cycle from the reference initial conditions and parameter values given in the original papers (S1 Text). They also exhibit phase-locking in response to pulse stimulation, in a similar way to the cells in Fig 2B. We wanted to develop suitable forms of interrogation that could discriminate between these models, but, in view of the cell-to-cell variation in response, we wanted to avoid fitting the models to data. Fitting to the population average data would not be meaningful, given such oscillatory behaviour, as no cell would behave like the average, and fitting to the data from any “representative” cell, however that cell might be chosen, runs the risk of overfitting, or capturing what is unique to that cell instead of what is general to all cells. We therefore developed a form of nonlinear frequency analysis that allows for nonstationarity, which, when coupled to parameter variation, enabled us to rule out three of the models. We then developed a nonlinear amplitude analysis that similarly ruled out three further models, leaving only the models AT1 and LR1, which passed both interrogations. These two models are schematically identical (Fig 3) but differ in their mathematical details. The models yielded unexpected predictions that we confirmed experimentally and further analysis showed how subtle mathematical differences can markedly change the distribution of model responses (Discussion).

## Results

### Nonlinear frequency analysis

**Fig 4:**
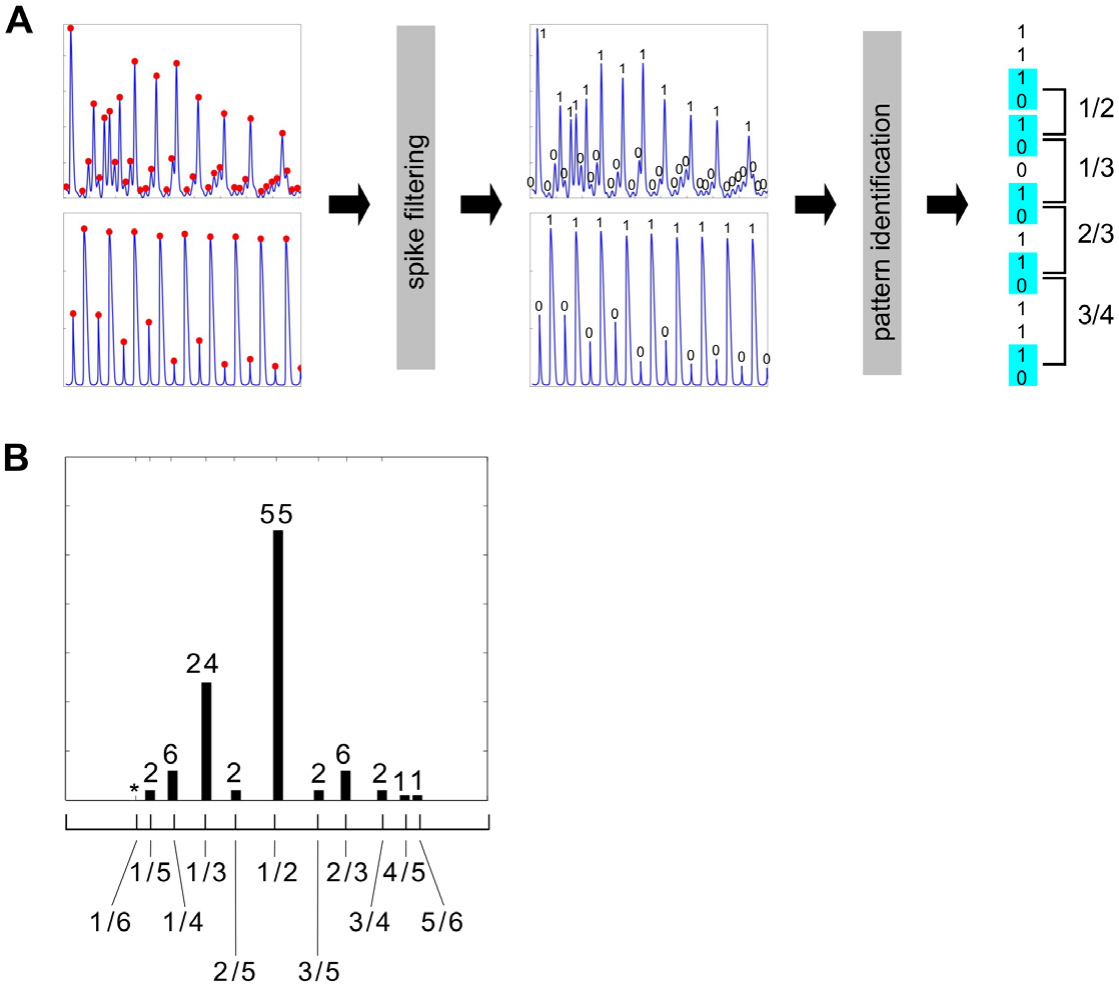
Method of nonlinear frequency analysis. **(A)** From left to right: experimental (above) and simulated (below) non-stationary Ca^2+^ time courses in response to pulse stimulation are processed by independent algorithms (S1 Text) to identify peaks in the data (red dots). A common “spike filtering” algorithm determines which peaks correspond to spikes (binary 1) or skips (binary 0), thereby generating a binary string. A “pattern identification” algorithm then locates each occurrence of the skipping indicator, “10”, in the binary string and determines the skipping pattern as the fraction of 1’s in the total number of binary digits before the next skipping indicator, as shown for a hypothetical bitstring on the right. **(B)** Experimental skipping-pattern data over all measured cells in twelve pulse stimulation experiments (experiment numbers 4–15 in S1 Text). The ticks beneath the panel mark the corresponding patterns according to the key below. The top boundary of the panel is 70% and the numbers over the bars are percentages to the nearest 1%, with an asterisk denoting a value below 1%.

To develop a nonlinear frequency analysis that allows for non-stationarity, we translated a phase-locked pattern of Ca^2+^ spikes into a bitstring by determining which peak in the data gave rise to a “spike” (binary 1) and which to a “skip” (binary 0) (Fig 4A). We then counted the skipping patterns between consecutive appearances of the bitstring “10”, which we took as the onset of a bout of skipping. A skipping pattern, represented as a fraction *i*/*n*, signifies *i* Ca^2+^ spikes out of *n* histamine pulses. The set of skipping patterns contains more information than a single time-averaged phase-locking ratio, as used previously [21], and better captures the heterogeneity of the response. Identical algorithms were applied, after spike identification, to experimental data from an individual cell and to the simulation output from each model, using the entire transient response from the onset of stimulation to incorporate the non-stationarity (Fig 4A).

Cell-to-cell variation in signalling responses are thought to arise primarily from extrinsic cell-to-cell variation in the concentrations of molecular components [24, 25]. As explained in the Introduction, we sought to exploit this additional information rather than average over it. We therefore aggregated the skipping pattern counts over all cells and over multiple experiments at different periods (Materials and Methods), to yield the skipping-pattern histogram in Fig 4B. The patterns 1/2 and 1/3 dominate, with more than half of all patterns being 1/2.

For a model, extrinsic variation between cells corresponds directly to variation in initial conditions (ICs) and also indirectly, through the influence of component concentrations on reaction rates, to variation in effective parameter values (PVs). Accordingly, for each model, we randomly selected sets of ICs and PVs at which the model exhibited oscillatory spiking in response to step stimulation. The empirical distributions of ICs and PVs are not well understood and measurements of them are difficult and largely lacking. In the absence of such data, we independently selected ICs and PVs using either uniform sampling or lognormal sampling around the reference values in the original papers (Materials and Methods).

**Fig 5:**
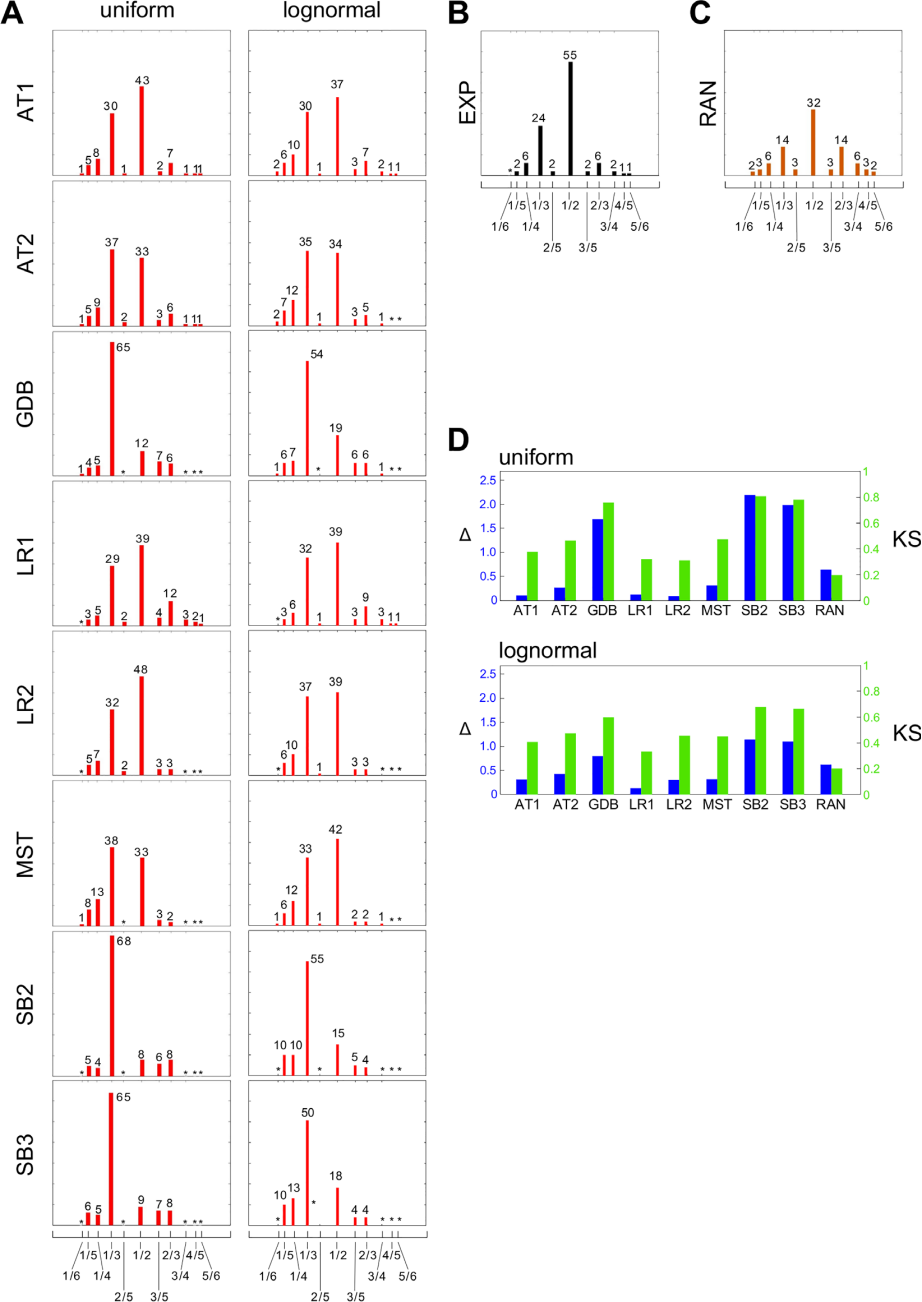
Nonlinear frequency analysis identifies classes of models. **(A)-(C)** Histograms of skipping-pattern frequencies, laid out as in Fig 4B and named on the left. **(A)** Model simulations (red), using the names in Fig 3, under uniform sampling (left column) and lognormal sampling (right column). **(B)** Experimental data (EXP, black) reproduced from Fig 4B for convenience of comparison. **(C)** Population of binary strings generated by independently choosing each bit at random with equal probability (RAN, gold). **(D)** Plots of the distance measure Δ (blue) and the KS statistic (green) (Materials and Methods) for uniform (top) and lognormal (bottom) sampling, showing the difference between the experimental distribution and each of the eight models and the random histogram, as annotated below the bars.

For each sampled set of ICs and PVs we determined the “natural period” of the corresponding model, as above for the experimental data. We then subjected each set of ICs and PVs to pulse stimulation at 20 different inter-pulse periods. Because inter-spike periods vary widely, we set the relative timescale of pulse to step stimulation in the middle of the experimental range, at 0.125. For each model, we aggregated the skipping-pattern counts over the sampled sets of ICs and PVs and all 20 stimulations (Fig 5A). We also calculated as a control the pattern histogram for a population of randomly chosen bitstrings. We found this to be markedly different from the simulated histograms in exhibiting symmetry around the 1/2 skipping pattern (Fig 5C).

The simulation histograms stabilise after several thousand samples and we chose 20,000 samples to provide a balance between coverage and efficiency. However, this number is still small in respect of the dimension of the space being sampled and we checked further for sampling errors. We undertook sub-sampling and super-sampling with both the uniform and the lognormal distributions. We found no evidence for heterogeneity (Figs J and K in S1 Text), except in the case of AT2.

Excluding AT2 for the moment, we found by inspection of Fig 5A three groups of histograms with slightly different membership of the groups depending on whether the sampling method was uniform or lognormal.

Under uniform sampling, GDB, SB2 and SB3 have high values at 1/3; MST has moderate values at 1/2 and 1/3, with 1/3 being the higher; and AT1, LR1 and LR2 also have moderate values at 1/2 and 1/3, with 1/2 being the higher. These qualitative relationships were all supported by sub- and super-sampling (Fig J in S1 Text). The last group best matched the experimental distribution (Fig 5B), as was further confirmed by two metrics, a distance measure, Δ, that we defined and the Kolmogorov–Smirnov (KS) statistic (Materials and Methods). When comparing histograms, it is important to use the metrics in conjunction with visual inspection, as histograms which are metrically close may still look quite different, as in the case of RAN.

Under lognormal sampling, MST still has moderate values at 1/2 and 1/3 but now 1/2 is higher, placing it in the same group as AT1, LR1 and LR2 which best matches EXP. The other group of GDB, SB2 and SB3 with high values at 1/3 remains the same. These qualitative relationships were also supported by sub- and super-sampling (Fig K in S1 Text). LR1 is now the closest to EXP under both metrics with AT1, LR2 and MST next closest.

The position of AT2 within these groups was ambiguous, with some samples showing 1/3 higher than 1/2 and some samples showing 1/2 higher than 1/3 (Figs J and K in S1 Text). It seemed unlikely that further sampling would decisively resolve this heterogeneity.

In summary, nonlinear frequency analysis rules out GDB, SB2 and SB3 and allows AT1, LR1 and LR2, under both uniform and lognormal sampling, while leaving AT2 and MST as possibilities under particular circumstances.

### Nonlinear amplitude analysis

Although nonlinear frequency analysis classified models into meaningful groups, it was unable to separate class 1 from class 2 models. In class 1, IP_3_ may initiate but does not drive Ca^2+^ oscillations, so that if IP_3_ oscillates, it does so only passively; in class 2, Ca^2+^ feeds back upon IP_3_, whose oscillation is thereby required for that of Ca^2+^ [22]. When HeLa cells respond to a step of histamine, IP_3_ has been found not to oscillate [26], indicating that whatever model is appropriate should be of class 1. The analysis correctly placed AT1 in the group closest to EXP but did not separate LR1 from LR2, while AT2 and MST are both class 2 and remain possibilities. In contrast to previous work [21], we find that phase-locking alone does not resolve key features of network architecture.

**Fig 6:**
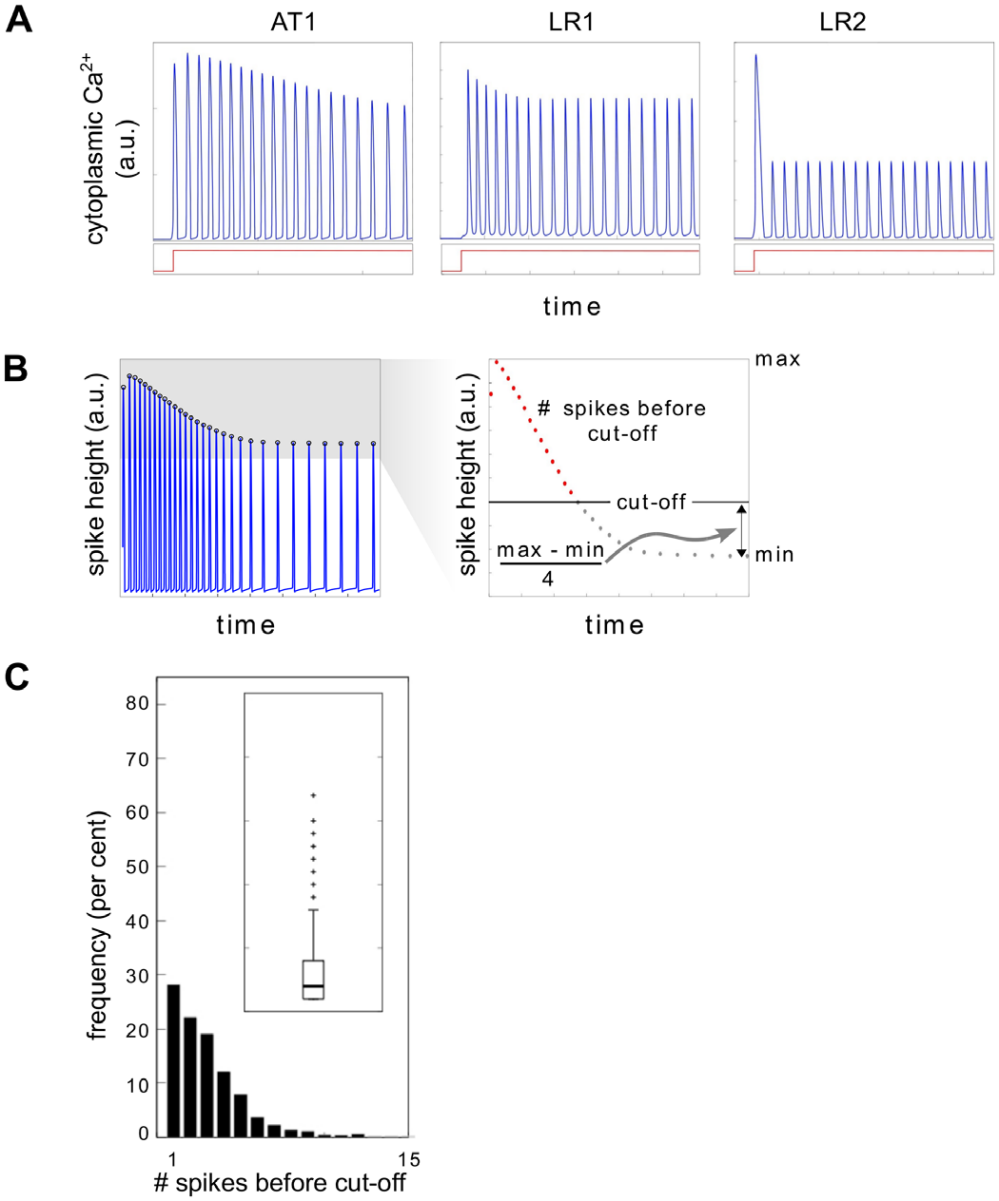
Method of nonlinear amplitude analysis. **(A)** Plots of cytoplasmic Ca^2+^ against time, in response to step stimulation, plotted underneath (red), for the AT1, LR1 and LR2 models, for the reference initial conditions (with initial Ca ER increased) and parameter values used in the original papers (S1 Text). **(B)** Measure of amplitude decay rate. Spike heights are plotted against time and the maximal (max) and minimal (min) heights over the time period are determined. A cut-off is set at the minimal height plus 1/4 of the difference between maximal and minimal. The number of spikes that occur before the cut-off is reached (red points) is taken as a measure of the rate at which amplitude decays. **(C)** Histogram of the amplitude decay rates over all measured cells in three step stimulation experiments (experiment numbers 1–3 in S1 Text). The inset shows a boxplot of the distribution, marked at the 25th percentile, median, 75th percentile, one standard deviation beyond the mean and outliers. The histogram is truncated at 15 spikes and the boxplot at 25 spikes.

We therefore sought further questions and turned to amplitude in place of frequency. The experimental response to step stimulation shows steadily decreasing spike amplitude after a high initial spike (Fig 2A). When initial Ca ER is increased from its reference value, we found similar behaviour in the AT1 model while LR2 settled immediately to a lower constant spike height and LR1 showed intermediate behaviour (Fig 6A). Accordingly, we defined a measure of amplitude decay rate based on counting the number of spikes before a specified cut-off (Fig 6B) and determined the histogram of this measure from all cells for all step stimulation experiments (Fig 6C). HeLa cells show a steady decrease in frequency of the number of spikes before cut-off.

**Fig 7:**
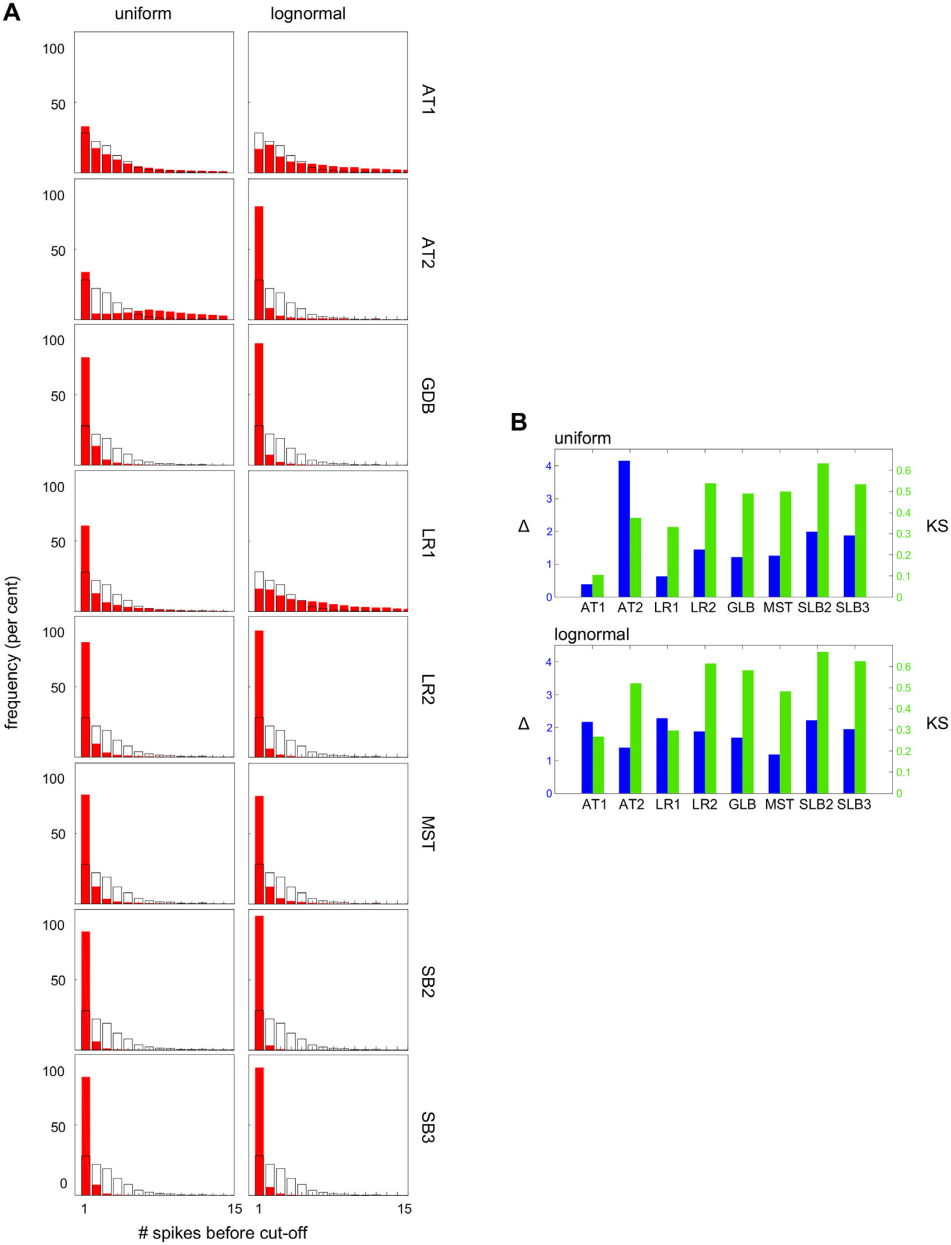
Amplitude analysis rules out class 2 models. **(A)** Histograms of the amplitude decay rates for all the models (for better comparison, a superimposed black contour of the experimental histogram in Fig 6C has been added to each panel). The histograms show the frequency with which a particular decay rate is found, under uniform (left) and lognormal (right) sampling of initial conditions and parameter values. **(B)** Distance measure Δ (left) and KS statistic (right) between the histograms of each of the 8 models and EXP for uniform (top) and lognormal (bottom) sampling.

We then calculated the distribution of this measure by simulation over all models under both uniform and lognormal sampling (Fig 7A). 20,000 samples gave stable distributions, with no exceptions found by sub- and super-sampling (Figs L and M in S1 Text). AT2, LR2 and MST were quite different by inspection from EXP under both uniform and lognormal sampling and this was supported by both metrics (Fig 7B). We were therefore able to rule out all the class 2 models which had survived nonlinear frequency analysis. As for the class 1 models, under uniform sampling, AT1 is a good match to EXP by inspection and is closest to EXP under both metrics, while LR1 is conspicuously worse. However, under lognormal sampling, AT1 and LR1 are the closest to EXP by visual inspection and their distance to EXP is almost the same under both metrics. Nonlinear amplitude analysis therefore leaves us with both AT1 and LR1 as possibilities.

### Different behaviour of AT1- and LR1-based hybrid models

AT1 and LR1 have identical schematics in Fig 3 but differ in the mathematical details of how components and feedbacks are represented. Their similarity makes it difficult to identify a third interrogation that would distinguish them. However, the analysis above does reveal a difference between the models: LR1, unlike AT1, shows a marked sensitivity to the sampling method (Fig 7A). We sought to understand how this sensitivity arises. We constructed 11 hybrid AT1-based models, as listed by number below, by starting with AT1 and replacing individual mathematical assumptions with those used in LR1.

1. SERCA pump. Replaced the hyperbolic function used in AT1 with the Hill function of coefficient 2 used in LR1.
2. IP_3_ receptor, Ca^2+^ -dependence. Replaced the hyperbolic function used in AT1 with the cubed hyperbolic function used in LR1.
3. IP_3_ receptor, IP_3_ -dependence. Replaced the linear function used in AT1 with the cubic function used in LR1.
4. IP_3_ receptor, proportion of open channels, which is treated as a dynamical variable in both models. Replaced the linear dependence in AT1 with the cubic dependence in LR1.
5. Combined the changes in models 2, 3 and 4 above.
6. Took the model in 5 above and further assumed that there was no leak current through the IP_3_ receptor from ER to cytoplasm, as there is in LR1.
7. IP_3_ receptor inactivation, Ca^2+^ -dependence. Replaced the Hill function of coefficient 2 in the AT1 model with the hyperbolic function in the LR1 model.
8. IP_3_ receptor, Ca^2+^ -independent term. Removed the basal flux in the AT1 model, which is not present in the LR1 model.
9. IP_3_ receptor, IP_3_ -independent term. Removed the basal flux as in model 8 above.
10. Combined the changes in models 8 and 9 above.
11. IP_3_ -independent leak current. Added this term, which is present in LR1.

We similarly constructed 11 LR1-based models by starting with LR1 and doing the opposite change to that listed above for models 1, 2, 4, 6, 7, 8, 9, 11 and the corresponding combination of changes as listed above for models 5, 10. Full details of these hybrid models are given in S1 Text. For the purposes of comparison, we also considered the unmodified AT1 (model 12), unmodified LR1 (model 13) and, as a further control, the original LR2 model (model 14).

**Fig 8:**
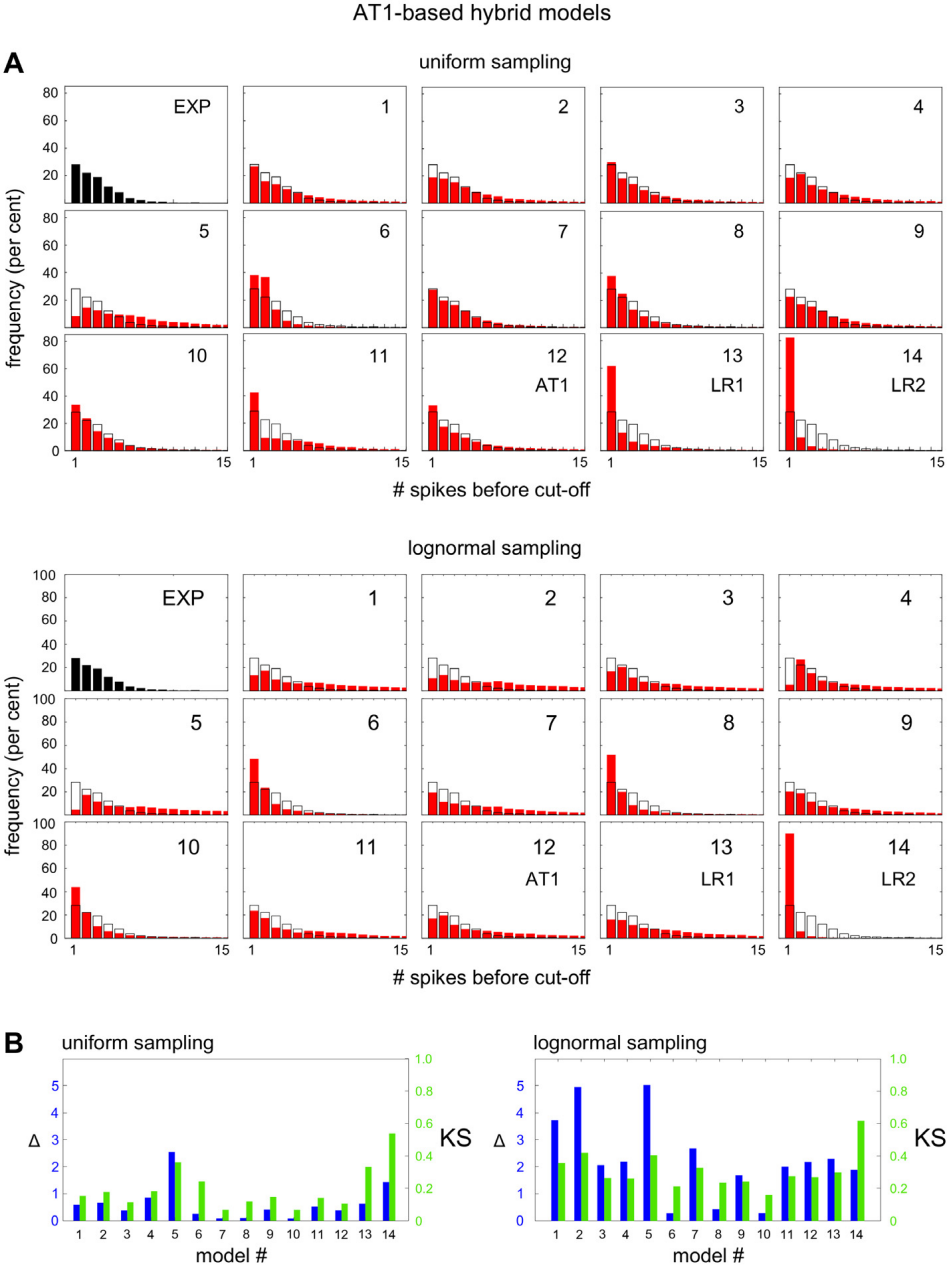
Amplitude analysis of hybrid AT1-based models. **(A)** Histograms of the amplitude decay rates, under uniform sampling (top) and under lognormal sampling (bottom), with a superimposed black contour of Fig 6C for easier comparison. Histograms 1–11 are from hybrid AT1 models, as described in the text, and histograms 12–14 are as annotated. **(B)** Metrics for the histograms in panel A.

**Fig 9:**
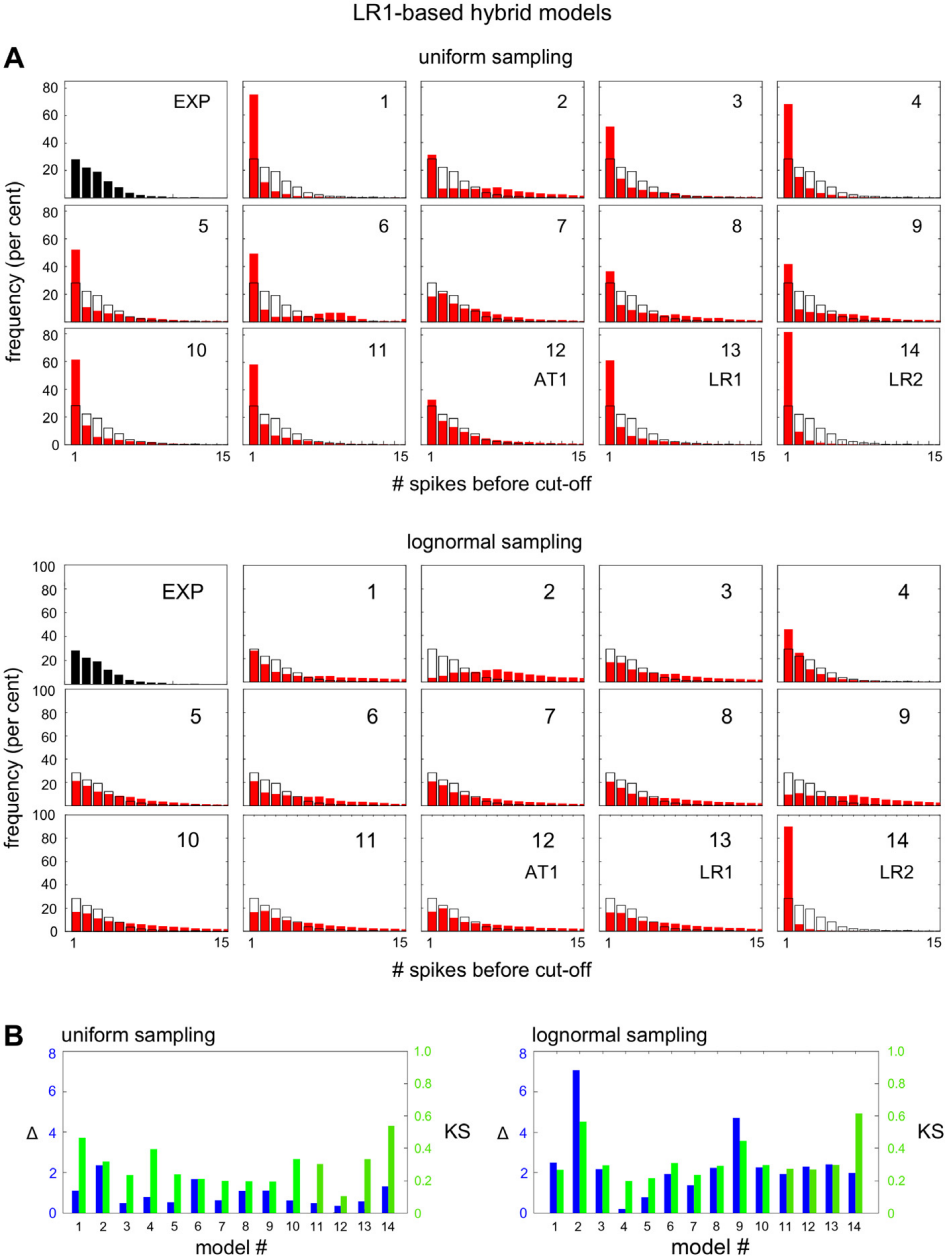
Amplitude analysis of hybrid LR1-based models. **(A)** Histograms of the amplitude decay rates for hybrid LR1-based models, under uniform sampling (top) and under lognormal sampling (bottom), laid out as in Fig 8. **(B)** Metrics for the histograms in panel A.

We subjected the AT1-based and LR1-based models to nonlinear amplitude analysis under both uniform and lognormal sampling and found a wide range of histograms (Figs 8A and 9A). Each individual model assumption has its own distinctive effect on the amplitude decay of the spiking behaviour. In particular, for the AT1-based models under uniform sampling, model 7 shows an excellent match, and the best match among all models, to the experimental data under visual inspection and both metrics, exceeding in quality that of AT1 itself. As for the sensitivity to the sampling method, all the LR1-based models, with one exception, exhibit similar sensitivity as LR1 itself (Fig 9A). The exception is also model 7. Model 7 concerns the Ca^2+^ -dependence of IP_3_ receptor inactivation, which changes from having a Hill coefficient of 2 in LR1 to a Hill coefficient of 1 in AT1. However, the loss of sampling sensitivity cannot be attributed to this feature in isolation, as the AT1-based model 7 does not acquire sampling sensitivity when the opposite change is made. The AT1-based models are generally as insensitive to the sampling method as AT1 itself (Fig 8A). We see that subtle mathematical details, as well as the choice of sampling method, can make a substantial difference to achieving a good match to the experimental data, confirming how difficult it can be to find the “correct” model when population variation is taken into account.

### AT1 and LR1 yield experimentally testable predictions

We sought predictions from the AT1 and LR1 models about HeLa cell responses. In these class 1 models, IP_3_ acts as a passive link between the input and the core oscillator (Fig 3) and decays exponentially when no histamine is present. If the gap between histamine pulses is large compared to the timescale of IP_3_ decay, IP_3_ should fall below the oscillator’s threshold and there should be no spike—indicating that the gap has been detected. Conversely, gaps that are small compared to the IP_3_ decay timescale should not be detected.

**Fig 10:**
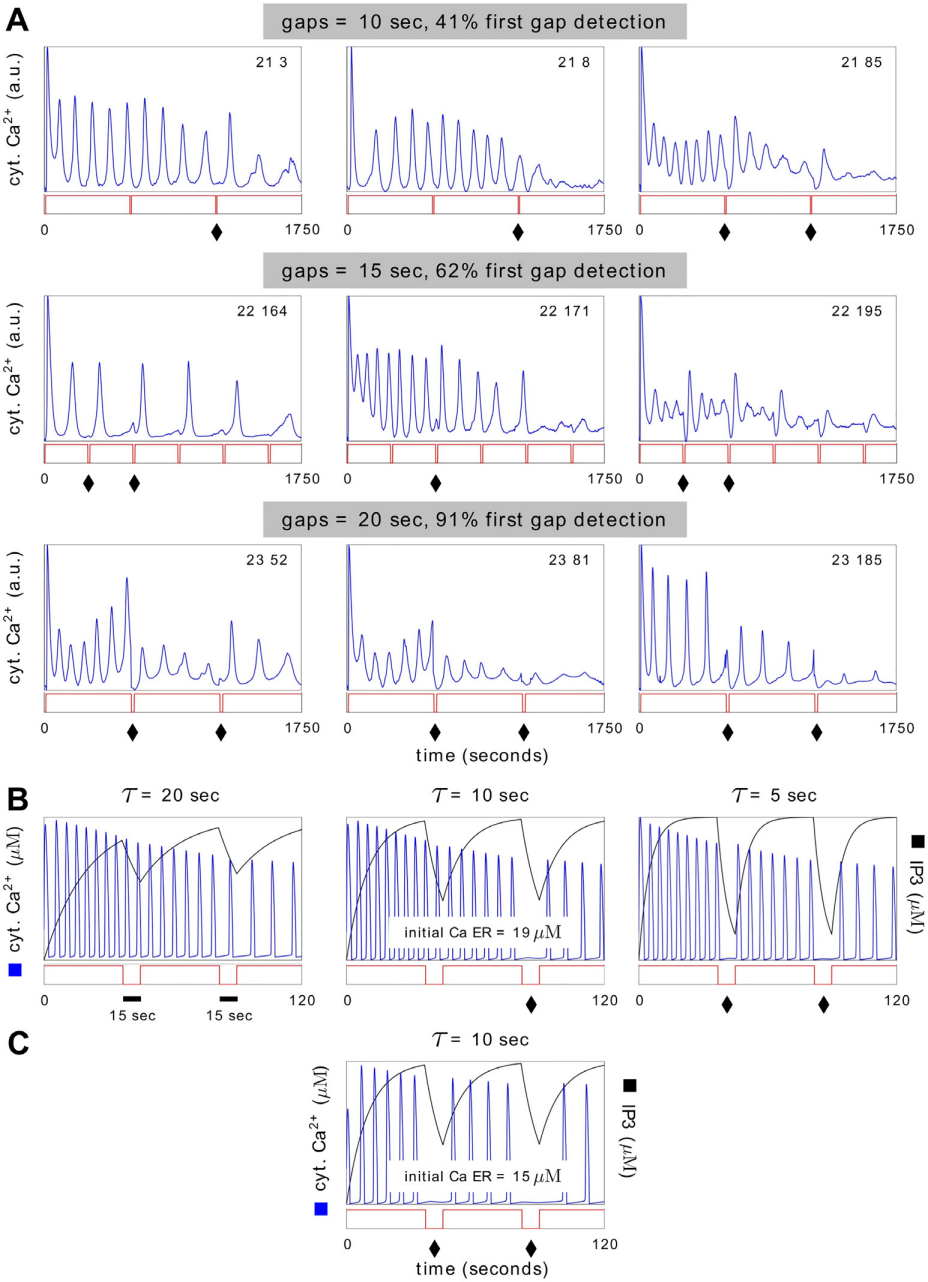
Gap detection with the AT1 model. **(A)** Plots of cytoplasmic Ca^2+^ for three representative cells in three experiments in which a step increase in histamine was interrupted by two (expt. 21 & 23) or five (expt. 22) gaps, with the size of the gap changing between experiments as shown. The first two gaps are marked by a black diamond when the gap was detected, based on a manual scoring of any feature of the Ca^2+^ trajectory that showed a discernible change at the gap (S1 Text). The percentage of cells that detect the first gap steadily increases with gap size but cells 21-3, 21-8 and 22-171 are able to detect the second gap despite missing the first. **(B)** Plots of cytoplasmic Ca^2+^ (blue) and IP_3_ (black) for the AT1 model responding to a step increase in histamine interrupted by two gaps of 15 seconds. The initial Ca ER and the timescale for IP_3_ decay, *τ*, were chosen as shown (*τ* corresponds to *ir*^−1^ in S1 Text), with the other initial conditions and parameter values as in the original paper. As *τ* decreases below the gap duration, the second gap is detected when *τ* = 10 sec and then both gaps are detected when *τ* = 5 sec. **(C)** The middle simulation in **B** is repeated with initial Ca ER lowered from 19 *μ*M to 15 *μ*M and both gaps are then detected.

Experiments with increasing gaps between histamine pulses showed a steadily increasing proportion of cells in which such gaps produce a detectable response (Fig 10A). The complexity of the spiking pattern makes it difficult to specify in computational terms whether or not a gap is detected but we found that manual scoring of the trend was perfectly consistent, with 5 out of 5 observers independently reporting an increase in cell proportions (Materials and Methods). Interestingly, we noticed that cells could sometimes detect a second inter-pulse gap without detecting the first. Both the AT1 model (Fig 10B) and the LR1 model reproduce this unexpected behaviour and further predict that the level of ER Ca^2+^ sets the detection threshold: for an IP_3_ decay timescale at which only the second gap is detected, decreasing just the initial ER Ca^2+^ allows both gaps to be detected (Fig 10C). The interplay between IP_3_ decay timescale and ER Ca^2+^ level in detecting inter-pulse gaps has also been reported for the response of HEK293 cells to carbachol [27, Fig 7].

## Discussion

There is a natural temptation to analyse cellular mechanisms “inside-out”, by intervening within cells and pulling the mechanisms apart, for which powerful tools exist in genetics, biochemistry and pharmacology. This approach has been less successful for understanding the networks underlying Ca^2+^ oscillation, perhaps because the Ca^2+^ signalling toolkit offers many alternative components to implement these networks in different cell types and because many different networks are capable of yielding such oscillations (Fig 3).

A complementary strategy is to recognise that the molecular complexity inside cells is, in some sense, a response over evolutionary time to the complexity of the environments in which those cells have existed, which has selected the information processing tasks that the cells have evolved to carry out. This raises the possibility of an “outside-in” strategy, in which the external environment is used to probe the internal molecular network [28].

Engineering offers several analogies for such an “outside-in” strategy. For instance, in communications engineering, it is well known that a linear system can be reconstructed from its frequency response. In a nonlinear biological system, the language of “bandwidth”, “filters” and “resonance”, based on measuring responses over a range of stimulation frequencies, can still be informative [9, 12], especially for homeostatic mechanisms close to their set points [10], where it may be reasonable to assume that the nonlinear system is well approximated by a linear one.

Another analogy is provided by the Internal Models Principle from control theory, which, in informal terms, states that if a system is to be controlled in such a manner that it is robust to some class of perturbations, then its controller must include a suitable model of those perturbations [29]. A particular example of this principle is the mechanism of integral feedback control, which, in the linear approximation, must be present if the system exhibits “perfect adaptation” to perturbation [30, 31]. Here, inferences are made about the internal mechanism (ie: the existence of integral feedback control) without intervening inside the cell. The idea of “internal models” has also been influential in neurobiology of motor control [32, 33]

A third engineering analogy lies in the Principle of Requisite Variety from cybernetics, which informally states that the information capacity of a system must match, in a suitable sense, that of its environment [34]. While resembling the justification given above for an “outside-in” strategy, this principle lacks an evolutionary motivation and has, so far, proved to be of limited biological value.

These analogies suggest that an outside-in strategy may be worth exploring further in systems biology but this has been hampered by several challenges. We know little about the actual spatio-temporal profile of the environments to which cells are exposed during development and physiology and it is difficult to manipulate such environments *in vivo*. It is also hard to reliably and reproducibly construct artificial environments and signals. Microfluidics has provided an important step forward in this respect, although, at the level of chemical signals, it remains difficult to reliably construct environmental signals that are more complicated than the pulse trains used here.

Nevertheless, further progress in this direction is likely and raises an interesting conceptual problem. What kinds of signals are most useful for discriminating models? For instance, if models are chosen from some class, perhaps defined by picking and mixing components in the manner of the calcium signalling toolkit [23], is it possible to define from among those signals that can be practically generated, a hierarchy of questions, which can efficiently discriminate one model from the others? Very little seems to be known about this problem but identifying a highly discriminatory class of signals could, in turn, encourage the development of microfluidic devices that can implement them.

The greatest challenge, of course, lies in the nature of living cells, which, among many other features, exhibit high degrees of nonlinearity, nonstationarity and heterogeneity. It has, accordingly, rarely been possible to adapt methods of engineering analysis directly. Instead, quantitative measures have to be defined that are appropriate to the particular biological context being studied, as was done here. For Ca^2+^ oscillation in response to hormone stimulation, the skipping phenomenon during phase-locking (Fig 2B) leads naturally to skipping pattern analysis (Fig 4A) while the amplitude decay in spiking (Fig 6A) leads naturally to the “spikes-before-cut-off” measure (Fig 6B). These measures appear reasonable for the spiking data that we found when we exposed HeLa cells to steps and pulses of histamine but other biological contexts may require different measures.

Crucially, for such quantitative measures to gain discriminatory power, it is essential to exploit cell-to-cell variation, as opposed to averaging over it. Each cell in the population is doing its own experiment in response to environmental stimulation and by accumulating this information, we can place greater constraints on the underlying model. We can do so, moreover, not by fitting the model to the data but, rather, by exploring its parametric sensitivity and matching that to what is found in the cell population. Exploiting cell-to-cell variation in this way distinguishes the methodology introduced here from previous efforts at developing “outside-in” strategies [10, 21].

The difficulty in exploiting population variation is that we lack empirical information about initial conditions and parameters. The latter, in particular, are usually “effective” parameters which may summarise complex mechanistic details. Their values are difficult to measure in individual cells in the first place, let alone over a population of cells, and very little is known about their distributions. We have adopted the tactic here of using two contrasting sampling methods, uniform and lognormal, and allowing models to succeed interrogation with either method. Although interrogation leads to two possible models, AT1 and LR1, these are so similar (Fig 3) that they give identical predictions which we were able to verify experimentally (Fig 10). We were also able to find differences between the models, in their sensitivity to uniform versus lognormal sampling (Figs 8 and 9), and to implicate the Ca^2+^ -dependence of IP_3_ receptor inactivation in this feature. However, this difference in behaviour cannot be readily exploited experimentally to distinguish the models.

The impact of cellular heterogeneity is particularly evident in the hybrid AT1-based and LR1-based models, which show distinctive changes in the distribution of amplitude decay rates to seemingly small changes in the mathematical details (Figs 8A and 9A). We see that finding the “correct” model for the cell type and hormone in question can be very difficult. We must emphasise that cellular interrogation may be able to discriminate between models but it cannot confirm that a model is the “correct” one. It is here that an “inside-out” strategy becomes invaluable; the two strategies, “outside-in” and “inside-out”, are not exclusive but complementary. We note further that a model is only “correct” in terms of the data against which it has been tested; there is no guarantee that it will remain correct if new data are acquired. As we have argued elsewhere [35], models are better seen as being “useful”, rather than as being “correct”, and it is in this spirit that the AT1 and LR1 models which emerges from interrogation should be viewed.

Oscillatory Ca^2+^ signalling has many advantages for exploring an “outside-in” strategy, not the least of which is that Ca^2+^ can be measured in single cells in real-time with considerable accuracy. However, oscillatory signalling is emerging as a much broader theme in biology [36–39]. Many other cellular components, such as key transcription factors, are now known to exhibit oscillations, to whose frequency downstream responses show differential sensitivity. It appears that evolution has exploited frequency modulation as a form of information processing, even when it is not a protection against toxicity. We hope that the methods introduced here for exploiting nonstationarity and heterogeneity may be usefully extended to these other biological systems. By asking cells the right questions, they may tell us more about themselves.

## Materials and Methods

### Microfluidic device fabrication

The microfluidic device was a simplified version of one developed in previous work [40]. Device construction uses soft lithography [17, 41], enabling automated fluid handling on the device through computer-controlled valves. The valves require two layers, each made from the elastomer polydimethylsiloxane (PDMS; Dow Corning, Midland, MI, USA), a control layer containing the control lines and a flow layer containing the flow lines. The PDMS layers are formed by replica molding from silicon masters on which features are constructed by standard photolithographic techniques. The two layers are bonded together to form the microfluidic device. Valves are created where a control line crosses a flow line, at which juncture a thin, flexible membrane separates the two lines. By increasing the fluid pressure in the control line, the membrane is deformed, thereby closing the valve and blocking the flow line. For proper closing, the flow line needs a semi-circular cross section. In our device, valves could be reliably opened and closed at a frequency of approximately 1Hz.

The masters for each layer were created by bonding photosensitive epoxy (a photoresist) to 3” silicon wafers. Patterns were created on the master by using photolithographic masks (Fig A in S1 Text) printed at 20,000dpi (CAD/Art Services Inc.) yielding 10 *μ*m minimum feature resolution.

The control layer master consisted of SU-8 photoresist (MicroChem, Newton, MA, USA) patterned into control lines 40 *μ*m thick using MicroChem’s SU-8 protocol (http://microchem.com/pdf/SU-82000DataSheet2025thru2075Ver4.pdf). The flow layer master required a two step process, with a layer of 22 *μ*m AZ 50XT photoresist (AZ Technology, Huntsville, AL, USA) for the flow lines, followed by a layer of 42 *μ*m SU-8 photoresist for the cell chamber and output port. (The cell chamber was cut off in the CIAD device, as explained below.) The three masks are shown in Fig A in S1 Text. The protocol for the AZ resist was adapted by us as follows.

The silicon wafer was etched in the plasma etcher at 115W for 5 minutes to loosen organic matter. The wafer was then spun at 1000 rpm, rinsed and swabbed twice with acetone, twice with methanol and twice with isopropanol. The wafer was then heated to 200°C for 5 minutes to dehydrate. The wafer was then covered with MCC primer (MicroChem, Newton, MA, USA) for 10 seconds, which was then spun off at 1000 rpm for 30 seconds and then baked at 100°C for 5 minutes. The AZ 50XT photoresist was carefully applied so as not to create air bubbles. The wafer was then spun at 2800 rpm for 30 seconds and then baked at 90°C at high humidity for 30 minutes. Humidity was achieved by placing beakers of water on the hotplate with the wafer and covering the entire assembly with aluminum foil. After baking, the wafer was placed in a 39% to 42% relative humidity chamber for 45 minutes, to equilibrate the water content of the photoresist to an optimal value. The wafer was exposed to UV light of 15 mW/cm^2^ constant intensity at 365 nm wavelength for 60 seconds, rested for 60 seconds, and then exposed again for 60 seconds. The wafer was developed in AZ400 developer (AZ Technology, Huntsville, AL, USA) diluted three parts water to one part developer and rinsed with deionised water. The temperature was ramped to 250°C over one hour to reflow and harden the resist. Reflow changes the cross-section of the resist from rectangular to semi-circular, which ensures proper closing of the flow lines during valve operation. The wafer was then cooled slowly to prevent cracking. The SU-8 photoresist was then applied following MicroChem’s SU-8 protocol, as above for the control layer master.

The flow layer itself was made by placing mixed and degassed PDMS, in the ratio 20:1 of pre-polymer to curing agent, on the flow layer master and spinning at 2100 rpm for 45 sec to achieve the desired thickness of 50 *μ*m. The control layer was made by placing mixed and degassed 5:1 PDMS on the control layer master inside a foil container for a resulting PDMS thickness of about 5mm. The flow and control assemblies were baked at 65°C for at least one hour. The control layer was then peeled from the master and cut into 6 individual pieces as marked by fiduciary lines. The pieces were then blown dry with nitrogen at 70 psig and bonded to the flow layer by plasma etching both pieces at 115 W, 115 mTorr for 15 sec and then baking the assembly at 65°C for one hour. The flow layer was carefully cut around the perimeter of the six control layer pieces using a curved-blade knife and peeled from the flow-layer master. Biopsy punches at 0.50 mm for control lines and 0.75 mm for flow lines were used to punch the holes in the devices at fiduciary marks in the PDMS. Razor blades were used to cut the devices to the desired size, indicated by line-shaped indentations in the PDMS. The devices were then rinsed with 100% isopropanol and blown dry with nitrogen. Other standard protocols, such as those for cleaning silicon wafers, may be found at (https://microfluidics.hms.harvard.edu/protocols.html).

### Chip-In-A-Dish (CIAD) fabrication and preparation

The device described above included a chamber for cells but we found that baseline Ca^2+^ measurements in the absence of hormone differed between cells grown on the device to those grown on a dish. We opted, therefore, to cut off the chamber from the device and to grow cells in a dish (Fig 1B), leaving the microfluidic device to undertake the fluid handling and pulse generation. A disadvantage of this design is attenuation and spreading of the pulse as it leaves the device output port. To counteract this, we measured Ca^2+^ responses only in those cells in the vicinity of the output port and used fluorescein dye to check that the cells in this region were receiving a properly-shaped pulse. We also determined skipping histograms in different areas of the dish and found no qualitative difference between them (Fig D in S1 Text).

To construct the CIAD, a microfluidic device fabricated as described above was plasma etched (115 W, 115 mTorr for 15 s) together with a 35 mm glass-bottom dish (In Vitro Scientific, D35-20-1-N) and the device was placed near the edge of the glass portion of the dish to bond. The whole assembly was then baked for at least 90 min at 65°C.

To prepare the CIAD for experiments, it was filled with Milli-Q purified water (EMD Millipore, Billerica, MA, USA) and placed under vacuum for at least 90 minutes to remove air from the control lines. Tygon tubing (Cole-Parmer, 06418-02) was cut to 25 cm lengths and connected to stainless steel tubes (New England Small tube, NE-1301-03) bent in half at a 90 degree angle. These were filled with water and the steel-tube end inserted into the control lines of the water-filled CIAD. The CIAD was then air dried and filled with collagen solution (Sigma, C8919, diluted with Milli-Q water 10:1), so as to also cover the glass portion of the dish. This was incubated for 24 hours, the collagen removed, the CIAD flushed with sterile water to remove excess collagen and vacuumed dry by inserting a syringe into the fluid lines and applying negative pressure for at least 10 seconds. Finally, the CIAD was sterilised by being placed under UV radiation inside a laminar flow cabinet for 24 hours.

To perform an experiment, water-filled control lines were connected to pneumatic lines at a pressure of 25 psig. These lines are controlled by a PCI card which is run by a purpose-built software program developed previously [40], which allows the valves to to be actuated to within millisecond time resolution. The two solutions used in the flow lines were 2.5 mM probenecid (Invitrogen, P36400) in HBSS (Sigma, H8264), with and without 10 *μM* histamine (Sigma, 53290), both pressurised to 5 psig (air pressure measured in fluid reservoir). The histamine valve and buffer valve were closed and the purge valves opened, allowing the two solutions to flow through their respective parts of the device. This process purges any air bubbles and remaining fluid from within the device and is done for 10 seconds or until all air bubbles are removed from the flow lines. At this point, the purge valves are closed.

### Tissue culture and experimental setup

HeLa Cells (ATCC, CCL-2, lot number 4965442) were incubated in T-25 flasks at 37°C with 5% CO_2_. They were passaged twice per week by trypsinizing the cells by rinsing with 5 mL HBSS and 1 mL trypsin EDTA (Cellgro, 25-052-Cl) and incubating for 5 minutes at 37°C in 5% CO_2_ and then splitting the suspension into three T-25 flasks, each with 3 mL to 5 mL media consisting of DMEM (Cellgro, 10-013-CV) with 10% FBS (Gibco, 26140) and 1% penicillin-streptomycin solution (100X, 10,000 I.U. penicillin, 10,000 μg/mL streptomycin (Cellgro, 30-002-CI)).

Confluent cells were trypsinized as described above and each CIAD was given 170 *μ*L cell suspension (approximately 4.7 × 10^5^ cells) with 1.83 mL media and 83 *μ*M trolox (Sigma, 238813-1G) as an antioxidant. This was incubated at 37°C in 5% CO_2_ for 24 hours.

To perform an experiment, a CIAD that had been incubated with cells, as above, was rinsed twice with 1 mL HBSS and then filled with 1 mL HBSS with 2.5 mM probenecid and 1 *μ*M Fluo-4 AM (Invitrogen, F14217) and left at room temperature (18°C to 20°C) for 45 minutes. Fluo-4 AM is a Ca^2+^ -sensitive, cell-permeant dye whose acetoxymethyl group is cleaved upon entering the cell, trapping the dye in the cytoplasm and excluding it from the mitrochondrion and other membrane-bound organelles. Probenecid reduces dye efflux from cells by inhibiting organic-anion transporters in the plasma membrane. The solution was then aspirated and the cells were soaked in HBSS with 2.5mM probenecid for another 45 minutes. During this period, the tubes leading to the flow lines were cleaned with ethanol and the appropriate histamine or buffer solution was run through the tubes to remove any remaining ethanol and air bubbles. The tubes were inserted into the CIAD and, after moving fluid through the device with the purge valves open for at least 10 seconds to remove air bubbles, the purge valves were closed. At the end of the 45 minute period, the device was imaged as described next.

### Imaging and image analysis

For imaging, we used a Zeiss Axiovert 135 TV inverted epi-fluorescence microscope equipped with a Chroma 49002 filter set. When bound to Ca^2+^, Fluo-4 has an absorption maximum at 494 nm and an emission maximum at 516 nm. We used an ET470/40x excitation filter, which covers a large part of Fluo-4’s absorption spectrum (although it does not overlap the absorption maximum) along with a T495lpxr dichroic mirror and ET525/50m emission filter, which cover Fluo-4’s emission spectrum very well. An HBO W/2 lamp was used as a broad-band light source. A Zeiss fluar 10x/0.5 M27 objective was chosen for its high transmittance in the range of Fluo-4’s absorbtion and emission wavelengths. A Hamamatsu C10600-10B camera provided a wide field of view and high spatial resolution, although, due to memory constraints, 4X binning was used during sampling. Micro-Manager 1.3, an open-source program developed by Ron Vale’s lab (http://valelab.ucsf.edu/~MM/MMwiki/index.php/Micro-Manager_Reader_as_ImageJ_plugin), which runs as a plugin to ImageJ [42], provided fully-automated control of the microscope for image acquisition. A 40 ms excitation UV pulse and a 10 ms exposure were used for each image, with images taken at a frame rate between 1 second/frame and 3 seconds/frame, so as to provide a minimum of 4 frames per inter-pulse gap. A low frame rate was used, relative to exposure time, to minimise phototoxicity and increase imaging time.

For each experiment, cells were segmented using the ImageJ macro in Fig B in S1 Text: all image frames were superimposed, thereby avoiding cells that were not adhered and, after background subtraction, the image was converted to a binary mask and segmented by the watershed algorithm. This created a template that defined the subsequent regions of interest (ROIs) in which fluorescence will be measured. Fluorescence integrated density—the product of the event area and the mean gray-scale value across the area—was measured in each frame for each ROI as a proxy for Ca^2+^ concentration in the cytoplasm, where the dye is localised.

#### Spike finding

The time series of Ca^2+^ intensities generated by ImageJ was further processed in Matlab. The time series was smoothed (*span* = 4) and a background correction applied, using a script developed by Vincent Mazet (http://www.mathworks.com/matlabcentral/fileexchange/27429-background-correction) [43]. For this method, based on visual fine tuning of multiple Ca^2+^ time series, we set *polynomial order* = 5 and *threshold* = 0.01 and chose an asymmetric Huber cost function [44]. The output from this procedure gave Ca^2+^ trajectories (Fig C in S1 Text), as shown in Fig 2A and 2B.

In a second processing step, Ca^2+^ spikes were identified in the trajectories. This was necessary to calculate the distribution of inter-spike periods in step-stimulation experiments (Fig 2A, inset), to determine whether or not to accept the data from a cell during pulse-stimulation experiments and to undertake nonlinear frequency analysis (Fig 4A). Spikes were located using an algorithm developed by Tom O’Haver and available as a Matlab routine from (http://terpconnect.umd.edu/~toh/spectrum/PeakFindingandMeasurement.htm). To reduce noise sensitivity in this method, we set *smooth width* = 4 and *fit width* = 4, so that only sufficiently broad spikes are detected, and no threshold was placed on the spike height (*threshold* = 0).

### Experiments undertaken

#### Step stimulation experiments

Three experiments were undertaken in which the cells were stimulated with a step function at 10*μ*M histamine concentration (Fig 2A). The experiments are listed below in Table 1, which shows for each experiment the number of cells that were segmented during the image analysis. These experiments provide the data for the inset in Fig 2A and for the the experimental histogram (EXP) in Fig 6C.

**Table 1:** Step stimulation experiments.

experiment	# segmented cells
1	286
2	249
3	535

For each cell in each of these three step experiments, Ca^2+^ spikes were located as described above and a mean inter-spike period was computed over the entire time course of observation. The distribution of these inter-spike periods over all cells in all three experiments (Fig 2A, inset) has a mean ± standard deviation of 130 ± 40 seconds.

#### Pulse stimulation experiments

Twelve experiments were undertaken in which the cells were subjected to repetitive pulses of 10*μ*M histamine (Paper Fig 2B), at a variety of inter-pulse periods and pulse widths. The experiments are listed in Table 2 below and provide the data from which Paper Fig 4B is aggregated. In each experiment a few cells appeared not to respond to the pulsing. To efficiently reject these, Ca^2+^ spikes were first located as described above and if the mean inter-spike period for a cell differed from the inter-pulse period by more than 30% of the mean, the cell was rejected. As shown in Table 2, this criterion was generous and the majority of cells were accepted.

**Table 2:** Pulse stimulation experiments.

experiment	period (sec)	width (sec)	# segmented cells	# accepted cells
4	40	15	108	98
5	40	15	153	137
6	60	22	575	449
7	20	5	460	417
8	60	22	568	461
9	60	38	299	229
10	20	5	649	595
11	40	10	461	247
12	40	10	402	334
13	40	25	367	366
14	60	38	364	219
15	60	38	379	276

For the model simulations discussed below, we wanted to choose pulse stimulations that bore a similar relationship to step stimulations at the same amplitude, as found in the experimental data. We used the ratio of the histamine pulse width to the average period between Ca^2+^ spikes. The latter quantity was reported above to be 130 seconds. As shown in Table 2, experimental pulse widths ranged from 5 seconds to 28 seconds, giving a timescale ratio between 5/130 (0.04) and 38/130 (0.29).

#### Gap experiments

Four experiments were undertaken with long pulses or, equivalently, with a step at 10 *μ*M histamine interrupted by gaps with no histamine. The experiments are listed in Table 3 below and provide the data from which the examples in Fig 10 are drawn. Experiments 16–20 have been omitted as they are not required for the results reported here. For each experiment, the Ca^2+^ trajectory from each segmented cell was manually scored as discussed below.

**Table 3:** Gap stimulation experiments. After a time of *t*_*hist*_ seconds, histamine is removed for a period of gap seconds and then added again. Two gaps were used in experiments 21 and 23 and five gaps in experiment 22.

experiment	*t*_*hist*_ (sec)	gap (sec)	# segmented cells
21	600	10	256
22	300	15	240
23	600	20	272

We found it difficult to develop a purely computational method of “gap detection” because of the extensive cell-to-cell variability and because so many different features of the trajectory looked as if they could be affected. We opted for manual scoring. Ca^2+^ trajectory plots for experiments 21–23, as listed above in Table 3, were provided to several of the authors, who independently scored them for the presence of any feature of the trajectories that appeared different at a gap. Experiment numbers were concealed and attention was focussed on the first gap. The results are tabulated below. There was substantial inter-personal variation in assessing whether or not a gap was detected, confirming the difficulty in identifying a computational measure of gap detection. However, each observer was consistent in reporting an increase in the number of detected gaps with increasing gap size. The values reported in Fig 10A are the average number of detected gaps as a percentage of the total number of cells, shown in the last column of Table 4.

**Table 4:** Manual scoring of first gap detection in experiments 21–23 in Table 3. The numbers in each column were independently reported by the author whose initials are given at the top of the column.

JE	NA	DG	FC	JG	Avg
experiment # 21 (256 cells)					
85	125	158	54	98	41%
experiment # 22 (240 cells)					
128	142	212	80	179	62%
experiment # 23 (272 cells)					
222	241	268	249	261	91%

### Mathematical modelling

Full details of the eight models which are schematically described in Fig 3 are provided in S1 Text. Because of the stiffness of the equations and the importance of numerical accuracy, we implemented our own 4th-order Runge-Kutta algorithm in Fortran for numerical simulation (available on request). The resulting trajectories, either for step or pulsed stimulation, are analysed in-situ and the results saved for later analysis using Matlab, as described further below.

### Nonlinear frequency analysis

#### Skipping pattern identification

We counted skipping patterns as follows. For the experimental data, for a specific choice of histamine pulse width and inter-pulse period as described in Table 2, and for each selected HeLa cell in the corresponding experiment, Ca^2+^ spikes were identified as described above. For a model simulation, given a specific choice of initial conditions (ICs) and parameter values (PVs) and a specific choice of input pulse height, pulse width and inter-pulse period, Ca^2+^ spikes were identified as described further below. Once spikes had been identified, whether in experimental data or simulation output, a common series of algorithms were used to identify patterns.

1. The series of spikes was first converted into a sequence of binary digits. A pseudo-code description of the *spikefilter* algorithm is shown on the left of Fig E in S1 Text. Briefly, a window of size plus or minus two spikes is moved over the sequence of spike heights. If the spike heights in the window have a large standard deviation compared to their mean and the spike at the center of the window is below that mean, then the centred spike is taken to be a skip (binary 0). All other spikes are taken to be full spikes (binary 1).
2. Skipping patterns were identified in the binary sequence, as described in Paper Fig 4A. The pseudo-code description of this *patternsearch* algorithm is shown on the right of Fig E in S1 Text.
3. Histograms of skipping pattern frequencies were collected, to yield the plots shown in Fig 5A, for simulations of the eight models, and (Figs 4B and 5B), for the data from experiments 4–15 in Table 2.

For reasons of computational efficiency, the *spikefilter* and *patternsearch* algorithms are performed during numerical simulation of the models, as described below, and were hence implemented in Fortran. For the experimental data, these same algorithms were implemented in Matlab.

#### Choosing input stimulations for the models

In comparing experimental data to model simulation, we seek to avoid fitting models to data. However, the numerical values for step and pulse stimulation of the models could not be directly calibrated against the values used experimentally, so we made the following choices to fix these numerical values.

1. For step stimulation of a model, we used the reference ICs and PVs in the original papers (S1 Text).
2. In each of the eight models, as described schematically in Fig 3, the input stimulation by hormone is treated as altering the value of one of the parameters, indicated by an asterisk, *, in the tables provided in S1 Text. Accordingly, the reference value of this parameter is the height of the step.
3. With this choice of step stimulation, as shown in the original papers, each model settles into a limit-cycle oscillation. By analogy with the experimental data (Fig 2A), the inter-spike period of this free-running oscillation was taken to be the *natural period* of the corresponding model.
4. For pulse stimulation of the model, the height of the pulse was set in the same way as with step stimulation, at the reference value of the input parameter. The pulse width was set so that the ratio of pulse width to the natural period of the model was 0.125 (= 1/8). This was chosen to lie in the middle of the corresponding range of experimental timescale ratios of 0.04 to 0.29, as for the pulse stimulation experiments described above. (We repeated the computational analysis at a timescale ratio of 0.25 and it did not alter our conclusions.)
5. Finally, the inter-pulse period was chosen as
  $$\frac{20\times \text{pulse}\;\text{width}}{i}\;,$$
  where *i* was varied from *i* = 1 to *i* = 19.

#### Choosing random ICs and PVs and defining the natural period

Step and pulse stimulation of a model depend on prior choice of numerical values for the ICs and PVs. We started from the reference values given in the tables in the S1 Text and then generated random distributions of ICs and PVs as described below. For each model, 20,000 sets of ICs and PVs are randomly chosen at which the corresponding model shows oscillatory behaviour in response to step stimulation, with the randomly chosen value of the input parameter being the height of the step, as described above. A flow chart and pseudo-code description of this algorithm are shown in Fig F in S1 Text. For reasons of efficiency the algorithm was implemented in Fortran in preference to Matlab.

In more detail, for uniform sampling, each IC and PV was taken to be *v* = *v*_0_ *r*, where *v*_0_ is the corresponding reference value (S1 Text) and *r* was independently drawn from the uniform distribution on [0, 10]; for lognormal sampling, each IC and PV was taken to be *v* = *v*_0_ *e*^*r*^, where *r* was independently drawn from the standard normal distribution. The ordinary differential equations in the model were then numerically integrated using a 4th-order Runge-Kutta solver, which yields a time series of Ca^2+^ levels. Simulations were undertaken for 2000 seconds.

The time series was examined to find time points at which the Ca^2+^ level is the largest of those lying in a window centered at the time point and having length plus or minus 4 time points. This was taken to be a spike. If 10 spikes were found in the time course, this was taken to be evidence that the time series was relaxing onto a limit cycle oscillation. This set of ICs and PVs were accepted as one of the 20,000 and the resulting mean inter-spike period was calculated as the *natural period* of the free running oscillator.

The repeated spikes that are found may reflect non-stationary behaviour prior to reaching the actual limit cycle. We chose to select oscillations and to calculate the natural period in this way so as to mimic the non-stationary behaviour found in the experimental data (Paper Fig 2A).

#### Pulse stimulation and pattern counting

For a set of randomly chosen ICs and PVs for which oscillation has been found and a natural period calculated, as previously, the model is further subjected to pulse stimulation and skipping patterns were counted in the resulting output, as described above. A flow chart and pseudo-code description of the algorithm are shown in Fig G in S1 Text.

Briefly, the pulse height, pulse width and inter-pulse period were chosen as described above. For each choice, the model was then numerically integrated with the same 4th order Runge-Kutta solver as previously but with a time varying input, corresponding to the repetitive pulses. A train of 30 pulses was used. Spikes were identified in the resulting output time series of Ca^2+^ levels, as described above, and the *spikefilter* and *patternsearch* algorithms, also described above, were called in turn. A series of 20 stimulations with varying inter-pulse periods was undertaken for each randomly chosen set of ICs and PVs and the resulting pattern frequencies were collected in the histograms in Fig 5A.

#### Histogram metrics

Cell-to-cell variation in experimental responses and parameter variation of models give rise to histograms (Figs 4B, 5A, 6C and 7A). We used two independent metrics to determine how close a model histogram, *m*, is to the corresponding experimental histogram, *e*. First, we defined a distance measure, Δ(*m*, *e*), using the formula

$$\Delta \left(m,e\right)=\sum_{i=1}^{K}\frac{{\left(m_{i}-e_{i}\right)}^{2}}{e_{i}}.$$

Here, *m*_*i*_ and *e*_*i*_ are expressed as probabilities, rather than percentages, and *K* is the number of bars in the histogram. We assume that bars are only measured when the experimental histogram has non-zero values, so that *e*_*i*_ ≠ 0 for 1 ≤ *i* ≤ *K* and Δ is well defined.

Second, we used the Kolmogorov-Smirnov statistic (KS) for discrete distributions [45]. The cumulative distribution functions for the model histogram, *m*, and the experimental histogram, *e*, were defined by

$$F_{m}\left(x\right)=\sum_{1\leq i\leq x}m_{i}\;,\;F_{e}\left(x\right)=\sum_{1\leq i\leq x}e_{i}$$

and the KS statistic was defined by

$$\text{KS}=\max_{x}\;\left|\;F_{e}\left(x\right)-F_{m}\left(x\right)\right|.$$

### Nonlinear amplitude analysis

In response to step stimulation, HeLa cells exhibit repetitive spiking with generally decreasing spike amplitude (Fig 2A). This decrease in amplitude is not due to photobleaching. We subjected cells to two step functions of histamine with a gap of 10 minutes between the steps and found that individual cells responded to the second step much as they responded to the first step (Fig H in S1 Text), suggesting that the decrease in amplitude is due to the dynamics of the molecular circuitry.

A measure of spike amplitude decay rate was defined (Fig 6B) and was applied to both experimental data and simulation output.

The experimental data was taken from the three step stimulation experiments in Table 1. For each selected cell in each experiment, spikes were identified as described above and the amplitude decay rate estimated. The distribution of decay rates is shown in Fig 6C.

For each of the models, 20,000 sets of ICs and PVs were randomly chosen, following the same procedure as described above for nonlinear frequency analysis. However, in addition to requiring oscillatory behaviour, the spike amplitudes were also required to decrease monotonically once the highest spike in the trajectory was reached. A flow chart and pseudo-code of this algorithm are shown in Fig I in S1 Text. The distribution of decay rates for all models over all sets of ICs and PVs is shown in Fig 7A.

## Supporting Information

S1 Text Mathematical descriptions of the models of oscillatory Ca^2+^ spiking, including hybrid AT1-LR1 models. Supplementary figures: (A) microfluidic device masks and setup; (B) macro used to segment cells; (C) segmentation process, from images to spiking trajectory; (D) heterogeneity control for CIAD imaging; (E) pseudo-code of the spike filtering and pattern search algorithms; (F) flow chart and pseudo-code for choosing random ICs and PVs and determining the natural period; (G) flow chart and pseudo-code for choosing random ICs and PVs and determining the natural period for pulse stimulation and pattern counting; (H) trajectories showing absence of photobleaching; (I) flow chart and pseudo-code for nonlinear amplitude analysis; (J) effect of sample size on nonlinear frequency analysis under uniform sampling; (K) effect of sample size on nonlinear frequency analysis under lognormal sampling; (L) effect of sample size on nonlinear amplitude analysis under uniform sampling; (M) effect of sample size on nonlinear amplitude analysis under lognormal sampling.
(PDF)

S1 Dataset Dataset for experiment 01. Each dataset provided as Supporting Information is an ASCII text file named “dataNN.zip”, where NN is the experiment number, covering 18 experiments numbered 01–15 and 21–23. The format of each file is as follows. Each line contains numbers separated by tabs and corresponds to a single time frame. The first number on a line is the number of the time frame. The remaining numbers on a line correspond to data obtained for each cell in the experiment at that time frame. The data for each cell are grouped into blocks of 7 numbers. If *cell*# denotes the number of the cell, counting in order along the line, and *B* = 7 * (*cell*# − 1), then the columns within each block are as follows.
column *B* + 2 is the *area in pixels*column *B* + 3 is the *mean grayscale pixel value*column *B* + 4 is the *standard deviation of pixel values*column *B* + 5 is the *X-coordinate of centre of mass in pixels*column *B* + 6 is the *Y-coordinate of centre of mass in pixels*column *B* + 7 is the *perimeter in pixels*column *B* + 8 is the *integrated density = sum of pixel values*
As an example, the experimental time-course data shown in Figs 2 or 10 corresponds to column 7 * (*cell*# − 1) + 3 in the compressed file “dataNN.zip”, where NN is the experiment number. The experiment number and the cell number are annotated in the top right corner of each figure panel.
(ZIP)

S2 Dataset Dataset for experiment 02. (ZIP)

S3 Dataset Dataset for experiment 03. (ZIP)

S4 Dataset Dataset for experiment 04. (ZIP)

S5 Dataset Dataset for experiment 05. (ZIP)

S6 Dataset Dataset for experiment 06. (ZIP)

S7 Dataset Dataset for experiment 07. (ZIP)

S8 Dataset Dataset for experiment 08. (ZIP)

S9 Dataset Dataset for experiment 09. (ZIP)

S10 Dataset Dataset for experiment 10. (ZIP)

S11 Dataset Dataset for experiment 11. (ZIP)

S12 Dataset Dataset for experiment 12. (ZIP)

S13 Dataset Dataset for experiment 13. (ZIP)

S14 Dataset Dataset for experiment 14. (ZIP)

S15 Dataset Dataset for experiment 15. (ZIP)

S16 Dataset Dataset for experiment 21. (ZIP)

S17 Dataset Dataset for experiment 22. (ZIP)

S18 Dataset Dataset for experiment 23. (ZIP)
