# Supplementary material for: Cellular Interrogation: Exploiting Cell-to-Cell Variability to Discriminate Regulatory Mechanisms in Oscillatory Signalling
Source: PLoS Comput Biol. 2016 Jul 1;12(7):e1004995. doi: 10.1371/journal.pcbi.1004995 (PMC4930170; doi:10.1371/journal.pcbi.1004995)
Supplement: S1 Text — Supplementary figures: (A) microfluidic device masks and setup; (B) macro used to segment cells; (C) segmentation process, from images to spiking trajectory; (D) heterogeneity control for CIAD imaging; (E) pseudo-code of the spike filtering and pattern search algorithms; (F) flow chart and pseudo-code for choosing random ICs and PVs and determining the natural period; (G) flow chart and pseudo-code for choosing random ICs and PVs and determining the natural period for pulse stimulation and pattern counting; (H) trajectories showing absence of photobleaching; (I) flow chart and pseudo-code for nonlinear amplitude analysis; (J) effect of sample size on nonlinear frequency analysis under uniform sampling; (K) effect of sample size on nonlinear frequency analysis under lognormal sampling; (L) effect of sample size on nonlinear amplitude analysis under uniform sampling; (M) effect of sample size on nonlinear amplitude analysis under lognormal sampling. (PDF) [file pcbi.1004995.s001.pdf]

SUPPORTING INFORMATION

Javier Estrada[1,*], Natalie Andrew[2,*], Daniel Gibson[4], Frederick Chang[3], Florian Gnad[4,†], Jeremy Gunawardena[4,§]

[1]Departamento de Física de la Materia Condensada and Instituto Nicolás Cabrera, Universidad Autónoma de Madrid, Spain.
[2]Department of Organismic and Evolutionary Biology, Harvard University, Cambridge, MA, USA.
[3]Molecular and Cell Biology Graduate Program, Harvard University, Cambridge, MA, USA.
[4]Department of Systems Biology, Harvard Medical School, Boston, MA, USA.

[*]These authors contributed equally.
[†]Current address: Genentech Inc, South San Francisco, CA, USA.
[§]Corresponding author; E-mail: jeremy@hms.harvard.edu

Abstract

This note provides supporting information for *"Cellular interrogation: exploiting cell-to-cell variability to discriminate regulatory mechanisms in oscillatory signalling"*. It should be read in conjunction with the paper, which provides further details. Software routines used in the paper are available on request.

Contents

1 Models of oscillatory Ca^{2+} spiking **1**
1.1 General comments about the models . . . 1
1.2 Meyer-Stryer—MST . . . 1
1.3 Goldbeter-Dupont-Berridge—GDB . . . 3
1.4 Atri *et al* class 1—AT1 . . . 4
1.5 Atri *et al* class 2—AT2 . . . 6
1.6 Li-Rinzel class 1—LR1 . . . 8
1.7 Li-Rinzel class 2—LR2 . . . 10
1.8 Sneyd-LeBeau two and three state—SB2 and SB3 . . . 12
1.9 Hybrid AT1-based and LR1-based models . . . 14

2 Supplementary Figures **27**

Microfluidic device and Chip-In-A-Dish. **28**

ImageJ macro used for segmentation of cells after imaging. **28**

Image segmentation and Ca^{2+} trajectory generation. **29**

Heterogeneity control for CIAD imaging. **29**

Pseudo-code of the spike filtering and pattern search algorithms. **30**

Flow chart and pseudo-code for choosing random ICs and PVs and determining the natural period. **31**

Flow chart and pseudo-code for pattern counting. **32**

Absence of photobleaching. **33**

Flow chart and pseudo-code for nonlinear amplitude analysis. **34**

Effect of sample size on nonlinear frequency analysis under uniform sampling **35**

Effect of sample size on nonlinear frequency analysis under lognormal sampling **36**

Effect of sample size on nonlinear amplitude analysis under uniform sampling **37**

Effect of sample size on nonlinear amplitude analysis under lognormal sampling **38**

1 Models of oscillatory Ca^{2+} spiking

1.1 General comments about the models

Eight models of oscillatory Ca^{2+} spiking were selected from the literature to cover a range of behaviours, as described in the Paper. The dynamical variables used in the various models are listed in Table 1 below. The differential equations for the models are listed in the sub-sections that follow, using the abbreviations

species	variable	units
cytoplasmic calcium	cc	μM
ER calcium	cer	μM
IP_3	p	μM
fraction of active IP_3 receptors	n	$u.l.$
fraction of receptors in state x	x	$u.l.$
fraction of receptors in state y	y	$u.l.$
fraction of receptors in state y_2	$y_2 = x + y$	$u.l.$

Table 1: Dynamical variables and their symbols and units; *u.l.* signifies an unitless number.

in Paper Figure 3. These equations follow the model descriptions in the original papers. Also listed in the accompanying table are the reference parameter values used in the original paper, at which the model exhibits limit cycle oscillations in response to step stimulation.

In the original papers, an input stimulation by hormone is regarded as varying one of the parameter values, so that the corresponding term in the equations is effectively taken to aggregate the effects of the hormone receptor and any downstream signalling between the receptor and the mechanism in which the parameter appears. We did not want to modify the original models and thereby make it difficult to compare our work to previous studies of calcium oscillation. We therefore chose to use the same approach. The input parameter for each model is flagged with an asterisk, $*$, in the corresponding table of reference parameter values.

1.2 Meyer-Stryer—MST

This is from the 1988 paper by Meyer and Stryer, *"Molecular model for receptor-stimulated calcium spiking"* [4].

$$\begin{aligned}
\frac{dp}{dt} &= k_+(cc) - k_-(p) \\
\frac{dcer}{dt} &= J_2(cer, cc) - J_1(p, cer) \\
\frac{dcc}{dt} &= J_1(p, cer) - J_2(cer, cc) - mitochondria(cc)
\end{aligned}$$

$$
\begin{aligned}
k_+(cc) &= \frac{c_4 R}{cc + k_3} \cdot cc \\
k_-(p) &= c_5 \cdot p \\
J_1(p, cer) &= \frac{c_1 \cdot cer \cdot p^3}{(k_1 + p)^3} \\
J_2(cer, cc) &= \frac{c_2 \cdot cc^2}{(cc + k_2)^2} - c_3 \cdot cer^2 \\
mitochondria(cc) &= c_6 (\frac{cc}{c_7})^{3.3} - c_6
\end{aligned}
$$

parameter	original value
c_1	6.64 s^{-1}
k_1	0.1 μM
c_2	5 $\mu M s^{-1}$
k_2	0.15 μM
c_3	3.13$\cdot 10^{-5}$ $(\mu M s)^{-1}$
c_4	1 s^{-1}
k_3	1 μM
c_5	2 s^{-1}
c_6	0.5 $\mu M s^{-1}$
c_7	0.6 μM
R^*	0.31 μM

variable	init. cond.
p	0.04 μM
cer	40 μM
cc	0.1 μM

Table 2: MST reference parameter values (left) and initial conditions (right) from [4].

1.3 Goldbeter-Dupont-Berridge—GDB

This is from the 1990 paper by Goldbeter, Dupont and Berridge, *"Minimal model for signal-induced Ca2+ oscillations and for their frequency encoding through protein phosphorylation"* [2].

$$\begin{aligned} \frac{dcer}{dt} &= v_2(cc) - v_3(cc, cer) - k_f \cdot cer \\ \frac{dcc}{dt} &= v_0 + v_1 \cdot b - v_2(cc) + v_3(cc, cer) + k_f \cdot cer - k \cdot cc \end{aligned}$$

$$\begin{aligned} v_2(cc) &= V_{M2} \cdot \frac{cc^2}{k_2^2 + cc^2} \\ v_3(cc, cer) &= V_{M3} \cdot \frac{cer^2}{K_R^2 + cer^2} \cdot \frac{cc^4}{K_A^4 + cc^4} \end{aligned}$$

parameter	original value
v_0	1 $\mu M s^{-1}$
k	10 s^{-1}
k_f	1 s^{-1}
v_1^*	7.3 $\mu M s^{-1}$
V_{M2}	65 $\mu M s^{-1}$
V_{M3}	500 $\mu M s^{-1}$
k_2	1 μM
K_R	2 μM
K_A	0.9 μM
b	0.31 $u.l.$

variable	init. cond.
cer	0.25 μM
cc	1.5 μM

Table 3: GDB reference parameter values (left) and initial conditions (right) from [2]

1.4 Atri *et al* class 1—AT1

This is from the 1993 paper by Atri, Amundson, Clapham and Sneyd, *"A single-pool model for intracellular calcium oscillations and waves in the Xenopus laevis oocyte"* [1], with modifications for the class I/II distinction from the 2006 paper by Sneyd, Tsaneva-Atanasova, Reznikov, Bai, Sanderson and Yule, *"A method for determining the dependence of calcium oscillations on inositol trisphosphate oscillations"* [6].

$$\begin{aligned}
\frac{dn}{dt} &= \frac{1}{\tau_n} \cdot (n_{inf}(cc) - n) \\
\frac{dp}{dt} &= ir \cdot (p_{st} - p) \\
\frac{dcer}{dt} &= J_{cc} - J_{IP3} \\
\frac{dcc}{dt} &= J_{IP3} - J_{cc} + \delta(J_{in} - J_{out})
\end{aligned}$$

$$\begin{aligned}
J_{cc} &= \frac{\gamma_2 \cdot cc}{k_{\gamma_2} + cc} \\
J_{IP3} &= k_{flux}\mu(p) \cdot n \left(b + \frac{V_1 cc}{k_1 + cc}\right)(\gamma \cdot cer - cc) \\
J_{in} &= \beta_1 + \beta_2 \cdot p_{st} \\
J_{out} &= \frac{\gamma_1 cc^2}{k_{\gamma_1}^2 + cc^2} \\
n_{inf}(cc) &= 1 - \frac{cc^2}{k_2^2 + cc^2} \\
\mu(p) &= \mu_0 + \frac{\mu_1 \cdot p}{k_\mu + p}
\end{aligned}$$

parameter	original value
p^*_{st}	10 μM
β_1	1 $\mu M s^{-1}$
β_2	0.2 s^{-1}
ir	0.08 s^{-1}
k_{flux}	4.8 $\mu M s^{-1}$
δ	0.01 $u.l.$
γ_1	24 $\mu M s^{-1}$
γ_2	20 $\mu M s^{-1}$
k_{γ_1}	0.4 μM
k_{γ_2}	0.06 μM
γ	5.405 μM^{-1}
τ_n	2 s
k_2	0.7 μM
b	0.111 $u.l.$
V_1	0.889 $u.l.$
k_1	0.7 μM
k_μ	4 μM
μ_0	0.567 $u.l.$
μ_1	0.433 $u.l.$

variable	init. cond.
n	0.986 $u.l.$
p	0 μM
cer	3.915194 μM
cc	0.083406 μM

Table 4: AT1 reference parameter values (left) and initial conditions (right) from [6]

1.5 Atri *et al* class 2—AT2

This is also from the 1993 paper by Atri, Amundson, Clapham and Sneyd, *"A single-pool model for intracellular calcium oscillations and waves in the Xenopus laevis oocyte"* [1], with modifications for the class I/II distinction from the 2006 paper by Sneyd, Tsaneva-Atanasova, Reznikov, Bai, Sanderson and Yule, *"A method for determining the dependence of calcium oscillations on inositol trisphosphate oscillations"* [6].

$$\begin{aligned}
\frac{dp}{dt} &= v_4 \left[\frac{cc + (1-\alpha)k_4}{cc + k_4}\right] - ir \cdot p \\
\frac{dcer}{dt} &= J_{cc} - J_{IP3} \\
\frac{dcc}{dt} &= J_{IP3} - J_{cc} + \delta(J_{in} - J_{out})
\end{aligned}$$

$$\begin{aligned}
J_{cc} &= \frac{\gamma_2 \cdot cc}{k_{\gamma_2} + cc} \\
J_{IP3} &= k_{flux}\mu(p) \cdot n_{inf}(cc)\left(b + \frac{V_1 cc}{k_1 + cc}\right)(\gamma \cdot cer - cc) \\
J_{in} &= \beta_1 + \beta_2 \cdot p_{st} \\
J_{out} &= \frac{\gamma_1 cc^2}{k_{\gamma_1}^2 + cc^2} \\
n_{inf}(cc) &= 1 - \frac{cc^2}{k_2^2 + cc^2} \\
\mu(p) &= \mu_0 + \frac{\mu_1 \cdot p}{k_\mu + p}
\end{aligned}$$

parameter	original value
v_4^*	6 $\mu M s^{-1}$
β_1	1 $\mu M s^{-1}$
β_2	0.2 s^{-1}
k_{flux}	4.8 $\mu M s^{-1}$
α	0.97 $u.l.$
δ	0.01 $u.l.$
γ_1	24 $\mu M s^{-1}$
γ_2	20 $\mu M s^{-1}$
k_{γ_1}	0.4 μM
k_{γ_2}	0.06 μM
γ	5.405 μM^{-1}
τ_n	2 s
k_2	0.7 μM
b	0.111 $u.l.$
V_1	0.889 $u.l.$
k_1	0.7 μM
k_μ	4 μM
μ_0	0.567 $u.l.$
μ_1	0.433 $u.l.$
k_4	1.1 μM
ir	0.08 s^{-1}

variable	init. cond.
p	0 μM
cer	3.915194 μM
cc	0.083406 μM
μ_0 and b to J_{IP3}	

Table 5: AT2 reference parameter values (left) and initial conditions (right) from [6]

1.6 Li-Rinzel class 1—LR1

This is from the 1994 paper by Li and Rinzel, *"Equations for $InsP_3$ receptor-mediated $[Ca^{2+}]_i$ oscillations derived from a detailed kinetic model: a Hodgkin-Huxley like formalism"* [3], with modifications for the class I/II distinction from the 2006 paper by Sneyd, Tsaneva-Atanasova, Reznikov, Bai, Sanderson and Yule, *"A method for determining the dependence of calcium oscillations on inositol trisphosphate oscillations"* [6].

$$
\begin{aligned}
\frac{dn}{dt} &= A(K_d - (cc + K_d) \cdot n) \\
\frac{dp}{dt} &= ir \cdot (p_{st} - p) \\
\frac{dcer}{dt} &= J_{cc} - J_{IP3} - J_{noIP3} \\
\frac{dcc}{dt} &= J_{IP3} + J_{noIP3} - J_{cc} + eps \cdot (J_{in} - J_{out})
\end{aligned}
$$

$$
\begin{aligned}
J_{cc} &= \frac{V_e cc^2}{K_e^2 + cc^2} \\
J_{IP3} &= P\left(\frac{p \cdot n \cdot cc}{(p + K_i) \cdot (cc + K_a)}\right)^3 \cdot (\sigma^{-1} cer - cc) \\
J_{noIP3} &= L(\sigma^{-1} cer - cc) \\
J_{in} &= \alpha_1 + \alpha_2 \cdot p_{st} \\
J_{out} &= \frac{V_p cc^2}{K_p^2 + cc^2}
\end{aligned}
$$

parameter	original value
p^*_{st}	0.8 μM
α_1	1 $\mu M s^{-1}$
α_2	0.25 s^{-1}
eps	0.01 $u.l.$
ir	0.02 s^{-1}
L	9.25·10^{-3} s^{-1}
P	66.6 s^{-1}
K_i	1 μM
K_a	0.4 μM
V_e	1 $\mu M s^{-1}$
K_e	0.2 μM
A	0.5 s^{-1}
K_d	0.4 μM
σ	0.185 $u.l.$
V_p	5 $\mu M s^{-1}$
K_p	0.3 μM

variable	init. cond.
n	0 $u.l.$
p	1.4 μM
cer	0.696 μM
cc	0.15 μM

Table 6: LR1 reference parameter values (left) and initial conditions (right) from [6]

1.7 Li-Rinzel class 2—LR2

This is also from the 1994 paper by Li and Rinzel, *"Equations for InsP$_3$ receptor-mediated [Ca^{2+}]$_i$ oscillations derived from a detailed kinetic model: a Hodgkin-Huxley like formalism"* [3], with modifications for the class I/II distinction from the 2006 paper by Sneyd, Tsaneva-Atanasova, Reznikov, Bai, Sanderson and Yule, *"A method for determining the dependence of calcium oscillations on inositol trisphosphate oscillations"* [6].

$$\begin{aligned}
\frac{dp}{dt} &= v_4\left[\frac{cc+(1-\alpha)k_4}{cc+k_4}\right] - ir\cdot p \\
\frac{dcer}{dt} &= J_{cc} - J_{IP3} - J_{noIP3} \\
\frac{dcc}{dt} &= J_{IP3} + J_{noIP3} - J_{cc} + eps\cdot(J_{in} - J_{out})
\end{aligned}$$

$$\begin{aligned}
J_{cc} &= \frac{V_e cc^2}{K_e^2 + cc^2} \\
J_{IP3} &= P\left(\frac{p\cdot n(cc)\cdot cc}{(p+K_i)\cdot(cc+K_a)}\right)^3\cdot(\sigma^{-1}cer - cc) \\
J_{noIP3} &= L(\sigma^{-1}cer - cc) \\
J_{in} &= \alpha_1 + \alpha_2\cdot p_{st} \\
J_{out} &= \frac{V_p cc^2}{K_p^2 + cc^2} \\
n(cc) &= \frac{K_d}{cc+K_d}
\end{aligned}$$

parameter	original value
v_4^*	0.7 $\mu M s^{-1}$
α	0.97 $u.l.$
k_4	1.1 μM
ir	0.02 s^{-1}
α_1	1 $\mu M s^{-1}$
α_2	0.25 s^{-1}
eps	0.01 $u.l.$
L	9.25$\cdot 10^{-3}$ s^{-1}
P	66.6 s^{-1}
K_i	1 μM
K_a	0.4 μM
add μ_0 and b to J_{IP3} V_e	1 $\mu M s^{-1}$
K_e	0.2 μM
A	0.5 s^{-1}
K_d	0.4 μM
σ	0.185 $u.l.$
V_p	5 $\mu M s^{-1}$
K_p	0.3 μM

variable	init. cond.
p	1.4 μM
cer	0.696 μM
cc	0.15 μM

Table 7: LR2 reference parameter values (left) and initial conditions from [6]

1.8 Sneyd-LeBeau two and three state—SB2 and SB3

These are from the 2000 paper by Sneyd, LeBeau and Yule, *"Traveling waves of calcium in pancreatic acinar cells: model construction and bifurcation analysis"* [5].

2 state:

$$\begin{aligned}
\frac{dy_2}{dt} &= \phi_3(cc)(1-y_2) - \left(\frac{\phi_1(cc)\phi_2(cc)P}{\phi_1(cc)P+\phi_{-1}(cc)}\right)\cdot y_2 \\
\frac{dcc}{dt} &= k_f\left(\frac{Py_2\phi_1(cc)}{\phi_1(cc)P+\phi_{-1}(cc)}\right)^4 - \frac{V_p cc^2}{K_p^2+cc^2} + J_{leak}
\end{aligned}$$

3 state:

$$\begin{aligned}
\frac{dx}{dt} &= \phi_{-1}(cc)\cdot y - P\phi_1(cc)\cdot x + \phi_3(cc)(1-x-y) \\
\frac{dy}{dt} &= P\phi_1(cc)\cdot x - \phi_{-1}(cc)\cdot y - \phi_2(cc)y \\
\frac{dcc}{dt} &= k_f y^4 - \frac{V_p cc^2}{K_p^2+cc^2} + J_{leak}
\end{aligned}$$

$$\begin{aligned}
\phi_1(cc) &= \frac{(k_1R_1+r_2cc)}{R_1+cc} \\
\phi_{-1}(cc) &= \frac{(k_{-1}+r_{-2})R_3}{R_3+cc} \\
\phi_2(cc) &= \frac{(k_2R_3+r_4cc)}{R_3+cc} \\
\phi_3(cc) &= \frac{(k_3R_5+r_6cc)}{R_5+cc}
\end{aligned}$$

parameter	original value
R_1	6 μM
r_2	100 s^{-1}
R_3	50 μM
r_4	20 s^{-1}
R_5	1.6 μM
r_6	0 s^{-1}
r_{-2}	0 s^{-1}
k_1	0 s^{-1}
k_2	0.53 s^{-1}
k_3	1 s^{-1}
k_{-1}	0.88 s^{-1}
k_f	28 $\mu M s^{-1}$
V_p	1.2 $\mu M s^{-1}$
J_{leak}	0.2 $\mu M s^{-1}$
p K_p	0.18 μM
P^*	0.47 $u.l.$

	variable	init. cond.
SB2	y_2	0.846 $u.l.$
	cc	0.15 μM
SB3	x	0.4 $u.l.$
	y	0.446 $u.l.$
	cc	0.15 μM

Table 8: SB2 and SB3 reference parameter values (left) and initial conditions (right) from [5]

1.9 Hybrid AT1-based and LR1-based models

The 11 hybrid models are listed below, following the numbering scheme in the paper, with the AT1-based hybrid given first and the corrresponding LR1-based hybrid given second. The specific modification is explained and the modified term is marked with an asterisk.

Hybrid 1

- AT1: replaced the hyperbolic function in J_{cc} by a Hill function of coefficient 2.

$$\begin{aligned}\frac{dn}{dt} &= \frac{1}{\tau_n}\cdot(n_{inf}(cc)-n)\\ \frac{dp}{dt} &= ir\cdot(p_{st}-p)\\ \frac{dcer}{dt} &= J_{cc}-J_{IP3}\\ \frac{dcc}{dt} &= J_{IP3}-J_{cc}+\delta(J_{in}-J_{out})\end{aligned}$$

$$\begin{aligned}J_{cc}* &= \frac{\gamma_2\cdot cc^2}{k_{\gamma_2}+cc^2}\\ J_{IP3} &= k_{flux}\mu(p)\cdot n\left(b+\frac{V_1 cc}{k_1+cc}\right)(\gamma\cdot cer-cc)\\ J_{in} &= \beta_1+\beta_2\cdot p_{st}\\ J_{out} &= \frac{\gamma_1 cc^2}{k_{\gamma_1}^2+cc^2}\\ n_{inf}(cc) &= 1-\frac{cc^2}{k_2^2+cc^2}\\ \mu(p) &= \mu_0+\frac{\mu_1\cdot p}{k_\mu+p}\end{aligned}$$

- LR1 version: replaced the Hill function in J_{cc} by a hyperbolic one.

$$\begin{aligned}\frac{dn}{dt} &= A(K_d-(cc+K_d)\cdot n)\\ \frac{dp}{dt} &= ir\cdot(p_{st}-p)\\ \frac{dcer}{dt} &= J_{cc}-J_{IP3}-J_{noIP3}\\ \frac{dcc}{dt} &= J_{IP3}+J_{noIP3}-J_{cc}+eps\cdot(J_{in}-J_{out})\end{aligned}$$

$$
\begin{aligned}
J_{cc}* &= \frac{V_e cc}{K_e + cc} \\
J_{IP3} &= P\left(\frac{p \cdot n \cdot cc}{(p + K_i) \cdot (cc + K_a)}\right)^3 \cdot (\sigma^{-1}cer - cc) \\
J_{noIP3} &= L(\sigma^{-1}cer - cc) \\
J_{in} &= \alpha_1 + \alpha_2 \cdot p_{st} \\
J_{out} &= \frac{V_p cc^2}{K_p^2 + cc^2}
\end{aligned}
$$

Hybrid 2

Replaced hyperbolic dependence of J_{IP3} on cc by a cubic function as in LR1.

$$
\begin{aligned}
\frac{dn}{dt} &= \frac{1}{\tau_n} \cdot (n_{inf}(cc) - n) \\
\frac{dp}{dt} &= ir \cdot (p_{st} - p) \\
\frac{dcer}{dt} &= J_{cc} - J_{IP3} \\
\frac{dcc}{dt} &= J_{IP3} - J_{cc} + \delta(J_{in} - J_{out})
\end{aligned}
$$

$$
\begin{aligned}
J_{cc} &= \frac{\gamma_2 \cdot cc}{k_{\gamma_2} + cc} \\
J_{IP3}* &= k_{flux}\mu(p) \cdot n\left(b + \frac{V_1 cc}{k_1 + cc}\right)^3 (\gamma \cdot cer - cc) \\
J_{in} &= \beta_1 + \beta_2 \cdot p_{st} \\
J_{out} &= \frac{\gamma_1 cc^2}{k_{\gamma_1}^2 + cc^2} \\
n_{inf}(cc) &= 1 - \frac{cc^2}{k_2^2 + cc^2} \\
\mu(p) &= \mu_0 + \frac{\mu_1 \cdot p}{k_\mu + p}
\end{aligned}
$$

- LR1 version: replace the cubic dependence of J_{IP3} on cc by a linear one.

$$
\begin{aligned}
\frac{dn}{dt} &= A(K_d - (cc + K_d) \cdot n) \\
\frac{dp}{dt} &= ir \cdot (p_{st} - p) \\
\frac{dcer}{dt} &= J_{cc} - J_{IP3} - J_{noIP3} \\
\frac{dcc}{dt} &= J_{IP3} + J_{noIP3} - J_{cc} + eps \cdot (J_{in} - J_{out})
\end{aligned}
$$

$$
\begin{aligned}
J_{cc} &= \frac{V_e cc^2}{K_e^2 + cc^2} \\
J_{IP3*} &= P\left(\frac{p}{(p+K_i)}\right)^3 \left(\frac{cc}{(cc+K_a)}\right) n^3 \cdot (\sigma^{-1} cer - cc) \\
J_{noIP3} &= L(\sigma^{-1} cer - cc) \\
J_{in} &= \alpha_1 + \alpha_2 \cdot p_{st} \\
J_{out} &= \frac{V_p cc^2}{K_p^2 + cc^2}
\end{aligned}
$$

Hybrid 3

Did the same thing as in Hybrid 2 but for IP_3, using the term $\mu(p)^3$

$$
\begin{aligned}
\frac{dn}{dt} &= \frac{1}{\tau_n} \cdot (n_{inf}(cc) - n) \\
\frac{dp}{dt} &= ir \cdot (p_{st} - p) \\
\frac{dcer}{dt} &= J_{cc} - J_{IP3} \\
\frac{dcc}{dt} &= J_{IP3} - J_{cc} + \delta(J_{in} - J_{out})
\end{aligned}
$$

$$
\begin{aligned}
J_{cc} &= \frac{\gamma_2 \cdot cc}{k_{\gamma_2} + cc} \\
J_{IP3*} &= k_{flux} \mu(p)^3 \cdot n \left(b + \frac{V_1 cc}{k_1 + cc}\right) (\gamma \cdot cer - cc) \\
J_{in} &= \beta_1 + \beta_2 \cdot p_{st} \\
J_{out} &= \frac{\gamma_1 cc^2}{k_{\gamma_1}^2 + cc^2} \\
n_{inf}(cc) &= 1 - \frac{cc^2}{k_2^2 + cc^2} \\
\mu(p) &= \mu_0 + \frac{\mu_1 \cdot p}{k_\mu + p}
\end{aligned}
$$

- LR1 version: replace the cubic dependence of J_{IP3} on p by a linear one.

$$
\begin{aligned}
\frac{dn}{dt} &= A(K_d - (cc + K_d) \cdot n) \\
\frac{dp}{dt} &= ir \cdot (p_{st} - p) \\
\frac{dcer}{dt} &= J_{cc} - J_{IP3} - J_{noIP3} \\
\frac{dcc}{dt} &= J_{IP3} + J_{noIP3} - J_{cc} + eps \cdot (J_{in} - J_{out})
\end{aligned}
$$

$$
\begin{aligned}
J_{cc} &= \frac{V_e cc^2}{K_e^2 + cc^2} \\
J_{IP3}* &= P\left(\frac{p}{(p+K_i)}\right)\left(\frac{cc}{(cc+K_a)}\right)^3 n^3 \cdot (\sigma^{-1}cer - cc) \\
J_{noIP3} &= L(\sigma^{-1}cer - cc) \\
J_{in} &= \alpha_1 + \alpha_2 \cdot p_{st} \\
J_{out} &= \frac{V_p cc^2}{K_p^2 + cc^2}
\end{aligned}
$$

Hybrid 4

Replaced the linear dependence of J_{IP3} on n by a cubic one.

$$
\begin{aligned}
\frac{dn}{dt} &= \frac{1}{\tau_n} \cdot (n_{inf}(cc) - n) \\
\frac{dp}{dt} &= ir \cdot (p_{st} - p) \\
\frac{dcer}{dt} &= J_{cc} - J_{IP3} \\
\frac{dcc}{dt} &= J_{IP3} - J_{cc} + \delta(J_{in} - J_{out})
\end{aligned}
$$

$$
\begin{aligned}
J_{cc} &= \frac{\gamma_2 \cdot cc}{k_{\gamma_2} + cc} \\
J_{IP3}* &= k_{flux}\mu(p) \cdot n^3 \left(b + \frac{V_1 cc}{k_1 + cc}\right)(\gamma \cdot cer - cc) \\
J_{in} &= \beta_1 + \beta_2 \cdot p_{st} \\
J_{out} &= \frac{\gamma_1 cc^2}{k_{\gamma_1}^2 + cc^2} \\
n_{inf}(cc) &= 1 - \frac{cc^2}{k_2^2 + cc^2} \\
\mu(p) &= \mu_0 + \frac{\mu_1 \cdot p}{k_\mu + p}
\end{aligned}
$$

- LR1 version: replace the cubic dependence of J_{IP3} on n by a linear one.

$$
\begin{aligned}
\frac{dn}{dt} &= A(K_d - (cc + K_d) \cdot n) \\
\frac{dp}{dt} &= ir \cdot (p_{st} - p) \\
\frac{dcer}{dt} &= J_{cc} - J_{IP3} - J_{noIP3} \\
\frac{dcc}{dt} &= J_{IP3} + J_{noIP3} - J_{cc} + eps \cdot (J_{in} - J_{out})
\end{aligned}
$$

$$
\begin{aligned}
J_{cc} &= \frac{V_e cc^2}{K_e^2 + cc^2} \\
J_{IP3*} &= P\left(\frac{p}{(p+K_i)}\right)^3 \left(\frac{cc}{(cc+K_a)}\right)^3 n \cdot (\sigma^{-1} cer - cc) \\
J_{noIP3} &= L(\sigma^{-1} cer - cc) \\
J_{in} &= \alpha_1 + \alpha_2 \cdot p_{st} \\
J_{out} &= \frac{V_p cc^2}{K_p^2 + cc^2}
\end{aligned}
$$

Hybrid 5

- AT1: combine the changes in Hybrids 2, 3 and 4.

$$
\begin{aligned}
\frac{dn}{dt} &= \frac{1}{\tau_n} \cdot (n_{inf}(cc) - n) \\
\frac{dp}{dt} &= ir \cdot (p_{st} - p) \\
\frac{dcer}{dt} &= J_{cc} - J_{IP3} \\
\frac{dcc}{dt} &= J_{IP3} - J_{cc} + \delta(J_{in} - J_{out})
\end{aligned}
$$

$$
\begin{aligned}
J_{cc} &= \frac{\gamma_2 \cdot cc}{k_{\gamma_2} + cc} \\
J_{IP3*} &= k_{flux} \mu(p)^3 \cdot n^3 \left(b + \frac{V_1 cc}{k_1 + cc}\right)^3 (\gamma \cdot cer - cc) \\
J_{in} &= \beta_1 + \beta_2 \cdot p_{st} \\
J_{out} &= \frac{\gamma_1 cc^2}{k_{\gamma_1}^2 + cc^2} \\
n_{inf}(cc) &= 1 - \frac{cc^2}{k_2^2 + cc^2} \\
\mu(p) &= \mu_0 + \frac{\mu_1 \cdot p}{k_\mu + p}
\end{aligned}
$$

- LR1 version: combine the changes in Hybrids 2, 3 and 4.

$$
\begin{aligned}
\frac{dn}{dt} &= A(K_d - (cc + K_d) \cdot n) \\
\frac{dp}{dt} &= ir \cdot (p_{st} - p) \\
\frac{dcer}{dt} &= J_{cc} - J_{IP3} - J_{noIP3} \\
\frac{dcc}{dt} &= J_{IP3} + J_{noIP3} - J_{cc} + eps \cdot (J_{in} - J_{out})
\end{aligned}
$$

$$
\begin{aligned}
J_{cc} &= \frac{V_e cc^2}{K_e^2 + cc^2} \\
J_{IP3}* &= P\left(\frac{p}{(p+K_i)}\right)\left(\frac{cc}{(cc+K_a)}\right) n \cdot (\sigma^{-1} cer - cc) \\
J_{noIP3} &= L(\sigma^{-1} cer - cc) \\
J_{in} &= \alpha_1 + \alpha_2 \cdot p_{st} \\
J_{out} &= \frac{V_p cc^2}{K_p^2 + cc^2}
\end{aligned}
$$

Hybrid 6

- AT1 version: starting with Hybrid 5, set $b = 0$ and $\mu_0 = 0$

$$
\begin{aligned}
\frac{dn}{dt} &= \frac{1}{\tau_n} \cdot (n_{inf}(cc) - n) \\
\frac{dp}{dt} &= ir \cdot (p_{st} - p) \\
\frac{dcer}{dt} &= J_{cc} - J_{IP3} \\
\frac{dcc}{dt} &= J_{IP3} - J_{cc} + \delta(J_{in} - J_{out})
\end{aligned}
$$

$$
\begin{aligned}
J_{cc} &= \frac{\gamma_2 \cdot cc}{k_{\gamma_2} + cc} \\
J_{IP3}* &= k_{flux}\mu(p)^3 \cdot n^3 \left(\frac{V_1 cc}{k_1 + cc}\right)^3 (\gamma \cdot cer - cc) \\
J_{in} &= \beta_1 + \beta_2 \cdot p_{st} \\
J_{out} &= \frac{\gamma_1 cc^2}{k_{\gamma_1}^2 + cc^2} \\
n_{inf}(cc) &= 1 - \frac{cc^2}{k_2^2 + cc^2} \\
\mu(p)* &= \frac{\mu_1 \cdot p}{k_\mu + p}
\end{aligned}
$$

- LR1 version: starting with Hybrid 5, add μ_0 and b to J_{IP3}

$$
\begin{aligned}
\frac{dn}{dt} &= A(K_d - (cc + K_d) \cdot n) \\
\frac{dp}{dt} &= ir \cdot (p_{st} - p) \\
\frac{dcer}{dt} &= J_{cc} - J_{IP3} - J_{noIP3} \\
\frac{dcc}{dt} &= J_{IP3} + J_{noIP3} - J_{cc} + eps \cdot (J_{in} - J_{out})
\end{aligned}
$$

$$
\begin{aligned}
J_{cc} &= \frac{V_e cc^2}{K_e^2 + cc^2} \\
J_{IP3}* &= P\left(\mu_0 + \frac{p}{(p+K_i)}\right)\left(b + \frac{cc}{(cc+K_a)}\right) n \cdot (\sigma^{-1} cer - cc) \\
J_{noIP3} &= L(\sigma^{-1} cer - cc) \\
J_{in} &= \alpha_1 + \alpha_2 \cdot p_{st} \\
J_{out} &= \frac{V_p cc^2}{K_p^2 + cc^2}
\end{aligned}
$$

Hybrid 7

- AT1 version: assumed a hyperbolic function for $n_{inf}(cc)$

$$
\begin{aligned}
\frac{dn}{dt} &= \frac{1}{\tau_n} \cdot (n_{inf}(cc) - n) \\
\frac{dp}{dt} &= ir \cdot (p_{st} - p) \\
\frac{dcer}{dt} &= J_{cc} - J_{IP3} \\
\frac{dcc}{dt} &= J_{IP3} - J_{cc} + \delta(J_{in} - J_{out})
\end{aligned}
$$

$$
\begin{aligned}
J_{cc} &= \frac{\gamma_2 \cdot cc}{k_{\gamma_2} + cc} \\
J_{IP3} &= k_{flux}\mu(p) \cdot n \left(b + \frac{V_1 cc}{k_1 + cc}\right)(\gamma \cdot cer - cc) \\
J_{in} &= \beta_1 + \beta_2 \cdot p_{st} \\
J_{out} &= \frac{\gamma_1 cc^2}{k_{\gamma_1}^2 + cc^2} \\
n_{inf}(cc)* &= 1 - \frac{cc}{k_2 + cc} \\
\mu(p) &= \mu_0 + \frac{\mu_1 \cdot p}{k_\mu + p}
\end{aligned}
$$

- LR1 version: assumed a Hill function term in dn/dt

$$
\begin{aligned}
\frac{dn}{dt}* &= A((1 - \frac{cc^2}{K_d^2 + cc^2}) - n) \\
\frac{dp}{dt} &= ir \cdot (p_{st} - p) \\
\frac{dcer}{dt} &= J_{cc} - J_{IP3} - J_{noIP3} \\
\frac{dcc}{dt} &= J_{IP3} + J_{noIP3} - J_{cc} + eps \cdot (J_{in} - J_{out})
\end{aligned}
$$

$$
\begin{aligned}
J_{cc} &= \frac{V_e cc^2}{K_e^2 + cc^2} \\
J_{IP3} &= P\left(\frac{p \cdot n \cdot cc}{(p + K_i) \cdot (cc + K_a)}\right)^3 \cdot (\sigma^{-1} cer - cc) \\
J_{noIP3} &= L(\sigma^{-1} cer - cc) \\
J_{in} &= \alpha_1 + \alpha_2 \cdot p_{st} \\
J_{out} &= \frac{V_p cc^2}{K_p^2 + cc^2}
\end{aligned}
$$

Hybrid 8

- AT1 version: set $b = 0$

$$
\begin{aligned}
\frac{dn}{dt} &= \frac{1}{\tau_n} \cdot (n_{inf}(cc) - n) \\
\frac{dp}{dt} &= ir \cdot (p_{st} - p) \\
\frac{dcer}{dt} &= J_{cc} - J_{IP3} \\
\frac{dcc}{dt} &= J_{IP3} - J_{cc} + \delta(J_{in} - J_{out})
\end{aligned}
$$

$$
\begin{aligned}
J_{cc} &= \frac{\gamma_2 \cdot cc}{k_{\gamma_2} + cc} \\
J_{IP3}* &= k_{flux} \mu(p) \cdot n \left(\frac{V_1 cc}{k_1 + cc}\right) (\gamma \cdot cer - cc) \\
J_{in} &= \beta_1 + \beta_2 \cdot p_{st} \\
J_{out} &= \frac{\gamma_1 cc^2}{k_{\gamma_1}^2 + cc^2} \\
n_{inf}(cc) &= 1 - \frac{cc^2}{k_2^2 + cc^2} \\
\mu(p) &= \mu_0 + \frac{\mu_1 \cdot p}{k_\mu + p}
\end{aligned}
$$

- LR1 version: add μ_0 to J_{IP3}

$$
\begin{aligned}
\frac{dn}{dt} &= A(K_d - (cc + K_d) \cdot n) \\
\frac{dp}{dt} &= ir \cdot (p_{st} - p) \\
\frac{dcer}{dt} &= J_{cc} - J_{IP3} - J_{noIP3} \\
\frac{dcc}{dt} &= J_{IP3} + J_{noIP3} - J_{cc} + eps \cdot (J_{in} - J_{out})
\end{aligned}
$$

$$
\begin{aligned}
J_{cc} &= \frac{V_e cc^2}{K_e^2 + cc^2} \\
J_{IP3}* &= P\left(\mu_0 + \frac{p}{(p+K_i)}\right)^3 \left(\frac{cc}{(cc+K_a)}\right)^3 n^3 \cdot (\sigma^{-1} cer - cc) \\
J_{noIP3} &= L(\sigma^{-1} cer - cc) \\
J_{in} &= \alpha_1 + \alpha_2 \cdot p_{st} \\
J_{out} &= \frac{V_p cc^2}{K_p^2 + cc^2}
\end{aligned}
$$

Hybrid 9

- AT1 version: set $\mu_0 = 0$

$$
\begin{aligned}
\frac{dn}{dt} &= \frac{1}{\tau_n} \cdot (n_{inf}(cc) - n) \\
\frac{dp}{dt} &= ir \cdot (p_{st} - p) \\
\frac{dcer}{dt} &= J_{cc} - J_{IP3} \\
\frac{dcc}{dt} &= J_{IP3} - J_{cc} + \delta(J_{in} - J_{out})
\end{aligned}
$$

$$
\begin{aligned}
J_{cc} &= \frac{\gamma_2 \cdot cc}{k_{\gamma_2} + cc} \\
J_{IP3} &= k_{flux}\mu(p) \cdot n \left(b + \frac{V_1 cc}{k_1 + cc}\right) (\gamma \cdot cer - cc) \\
J_{in} &= \beta_1 + \beta_2 \cdot p_{st} \\
J_{out} &= \frac{\gamma_1 cc^2}{k_{\gamma_1}^2 + cc^2} \\
n_{inf}(cc) &= 1 - \frac{cc^2}{k_2^2 + cc^2} \\
\mu(p)* &= \frac{\mu_1 \cdot p}{k_\mu + p}
\end{aligned}
$$

- LR1 version: add b to J_{IP3}

$$
\begin{aligned}
\frac{dn}{dt} &= A(K_d - (cc + K_d) \cdot n) \\
\frac{dp}{dt} &= ir \cdot (p_{st} - p) \\
\frac{dcer}{dt} &= J_{cc} - J_{IP3} - J_{noIP3} \\
\frac{dcc}{dt} &= J_{IP3} + J_{noIP3} - J_{cc} + eps \cdot (J_{in} - J_{out})
\end{aligned}
$$

$$
\begin{aligned}
J_{cc} &= \frac{V_e cc^2}{K_e^2 + cc^2} \\
J_{IP3}* &= P\left(\frac{p}{(p+K_i)}\right)^3 \left(b + \frac{cc}{(cc+K_a)}\right)^3 n^3 \cdot (\sigma^{-1}cer - cc) \\
J_{noIP3} &= L(\sigma^{-1}cer - cc) \\
J_{in} &= \alpha_1 + \alpha_2 \cdot p_{st} \\
J_{out} &= \frac{V_p cc^2}{K_p^2 + cc^2}
\end{aligned}
$$

Hybrid 10

- AT1 version: set $b = 0$ and $\mu_0 = 0$

$$
\begin{aligned}
\frac{dn}{dt} &= \frac{1}{\tau_n} \cdot (n_{inf}(cc) - n) \\
\frac{dp}{dt} &= ir \cdot (p_{st} - p) \\
\frac{dcer}{dt} &= J_{cc} - J_{IP3} \\
\frac{dcc}{dt} &= J_{IP3} - J_{cc} + \delta(J_{in} - J_{out})
\end{aligned}
$$

$$
\begin{aligned}
J_{cc} &= \frac{\gamma_2 \cdot cc}{k_{\gamma_2} + cc} \\
J_{IP3}* &= k_{flux}\mu(p) \cdot n \left(\frac{V_1 cc}{k_1 + cc}\right) (\gamma \cdot cer - cc) \\
J_{in} &= \beta_1 + \beta_2 \cdot p_{st} \\
J_{out} &= \frac{\gamma_1 cc^2}{k_{\gamma_1}^2 + cc^2} \\
n_{inf}(cc) &= 1 - \frac{cc^2}{k_2^2 + cc^2} \\
\mu(p)* &= \frac{\mu_1 \cdot p}{k_\mu + p}
\end{aligned}
$$

- LR1 version: add μ_0 and b to J_{IP3}

$$
\begin{aligned}
\frac{dn}{dt} &= A(K_d - (cc + K_d) \cdot n) \\
\frac{dp}{dt} &= ir \cdot (p_{st} - p) \\
\frac{dcer}{dt} &= J_{cc} - J_{IP3} - J_{noIP3} \\
\frac{dcc}{dt} &= J_{IP3} + J_{noIP3} - J_{cc} + eps \cdot (J_{in} - J_{out})
\end{aligned}
$$

$$
\begin{aligned}
J_{cc} &= \frac{V_e cc^2}{K_e^2 + cc^2} \\
J_{IP3}* &= P\left(\mu_0 + \frac{p}{(p+K_i)}\right)^3 \left(b + \frac{cc}{(cc+K_a)}\right)^3 n^3 \cdot (\sigma^{-1} cer - cc) \\
J_{noIP3} &= L(\sigma^{-1} cer - cc) \\
J_{in} &= \alpha_1 + \alpha_2 \cdot p_{st} \\
J_{out} &= \frac{V_p cc^2}{K_p^2 + cc^2}
\end{aligned}
$$

Hybrid 11

- AT1 version: include $J_{noIP3} = L(\gamma \cdot cer - cc)$

$$
\begin{aligned}
\frac{dn}{dt} &= \frac{1}{\tau_n} \cdot (n_{inf}(cc) - n) \\
\frac{dp}{dt} &= ir \cdot (p_{st} - p) \\
\frac{dcer}{dt} &= J_{cc} - J_{IP3} - J_{noIP3} \\
\frac{dcc}{dt} &= J_{noIP3} + J_{IP3} - J_{cc} + \delta(J_{in} - J_{out})
\end{aligned}
$$

$$
\begin{aligned}
J_{cc} &= \frac{\gamma_2 \cdot cc}{k_{\gamma_2} + cc} \\
J_{IP3} &= k_{flux} \mu(p) \cdot n \left(b + \frac{V_1 cc}{k_1 + cc}\right) (\gamma \cdot cer - cc) \\
J_{noIP3}* &= L(\gamma \cdot cer - cc) \\
J_{in} &= \beta_1 + \beta_2 \cdot p_{st} \\
J_{out} &= \frac{\gamma_1 cc^2}{k_{\gamma_1}^2 + cc^2} \\
n_{inf}(cc) &= 1 - \frac{cc^2}{k_2^2 + cc^2} \\
\mu(p) &= \mu_0 + \frac{\mu_1 \cdot p}{k_\mu + p}
\end{aligned}
$$

- LR1 version: set $J_{noIP3} = 0$

$$\begin{aligned}
\frac{dn}{dt} &= A(K_d - (cc + K_d) \cdot n) \\
\frac{dp}{dt} &= ir \cdot (p_{st} - p) \\
\frac{dcer}{dt} &= J_{cc} - J_{IP3} - J_{noIP3} \\
\frac{dcc}{dt} &= J_{IP3} + J_{noIP3} - J_{cc} + eps \cdot (J_{in} - J_{out})
\end{aligned}$$

$$\begin{aligned}
J_{cc} &= \frac{V_e cc^2}{K_e^2 + cc^2} \\
J_{IP3} &= P \left(\frac{p \cdot n \cdot cc}{(p + K_i) \cdot (cc + K_a)} \right)^3 \cdot (\sigma^{-1} cer - cc) \\
J_{noIP3}* &= 0 \\
J_{in} &= \alpha_1 + \alpha_2 \cdot p_{st} \\
J_{out} &= \frac{V_p cc^2}{K_p^2 + cc^2}
\end{aligned}$$

References

[1] A. Atri, J. Amundson, D. Clapham, and J. Sneyd. A single-pool model for intracellular calcium oscillations and waves in the Xenopus laevis oocyte. *Biophys. J.*, 65:1727–39, 1993.

[2] A. Goldbeter, G. Dupont, and M. J. Berridge. Minimal model for signal-induced Ca2+ oscillations and for their frequency encoding through protein phosphorylation. *Proc. Natl. Acad. Sci. USA*, 87:1461–5, 1990.

[3] Y. X. Li and J. Rinzel. Equations for $InsP_3$ receptor-mediated $[Ca^{2+}]_i$ oscillations derived from a detailed kinetic model: a Hodgkin-Huxley like formalism. *J. Theor. Biol.*, 166:461–73, 1994.

[4] T. Meyer and L. Stryer. Molecular model for receptor-stimulated calcium spiking. *Proc. Natl. Acad. Sci. USA*, 85:5051–5, 1988.

[5] J. Sneyd, A. LeBeau, and D. Yule. Traveling waves of calcium in pancreatic acinar cells: model construction and bifurcation analysis. *Physica D*, 145:158–79, 2000.

[6] J. Sneyd, K. Tsaneva-Atanasova, V. Reznikov, Y. Bai, M. J. Sanderson, and D. I. Yule. A method for determining the dependence of calcium oscillations on inositol trisphosphate oscillations. *Proc. Natl. Acad. Sci. USA*, 103:1675–80, 2006.

2 Supplementary Figures

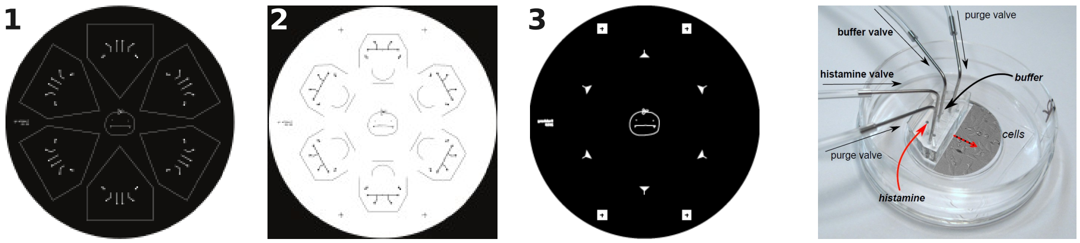

Figure A: Microfluidic device and Chip-In-A-Dish. Left, lithographic masks for the control layer (1) and the two flow layers (2,3), allowing six devices to be made in one operation. When the layers are bonded together, the lines in the control layer overlap with the lines in the flow layer to form the valves. Fiduciary marks are used to align the layers. Right, photograph of a Chip-In-A-Dish (CIAD) which is assembled by bonding a microfluidic device to a glass-bottomed dish, as also shown in Paper Figure 7. Histamine and buffer solutions are pumped in at 5 psig with tubes attached to the indicated holes (these tubes are not shown in the photograph). Valves are controlled by compressed air at 25 psig. With the purge valves closed, alternately opening and closing the buffer and histamine valves creates a series of histamine pulses that flow out of the device and stimulate the cells in the dish.

```
//Macro which selects cells from a UV stack and prepares measure over time: area,mean, st.dev and integral density and so on
//At the end, menu opens to save results in excel format

run("Z Project...", "start=1 stop=300 projection=[Max Intensity]");
run("Subtract Background...", "rolling=10");
run("Sharpen");
setAutoThreshold("Mean");
run("Convert to Mask");
run("Invert");
run("Watershed");
run("Analyze Particles...", "size=16-500 circularity=0.00-1.00 show=Outlines display clear include add");
run("Set Measurements...", "area mean standard integrated redirect=None decimal=0");
```

Figure B: ImageJ macro used for segmentation of cells after imaging.

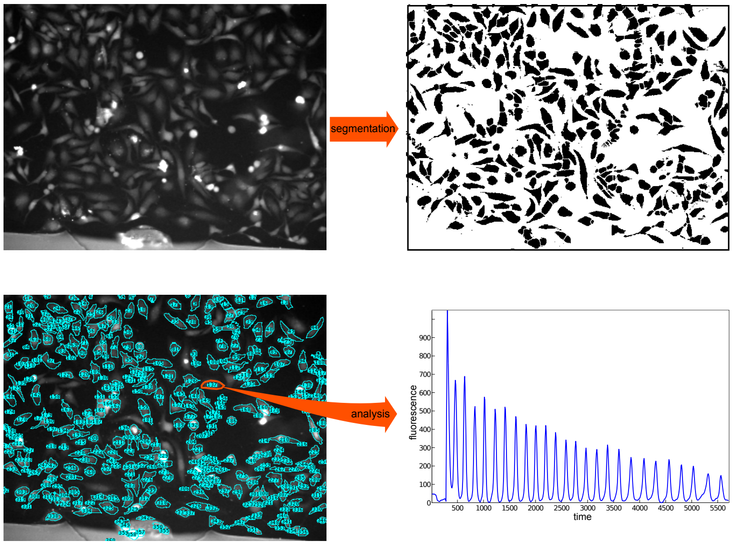

Figure C: Image segmentation and Ca^{2+} trajectory generation. All frames are superimposed (top left) to create a binary mask (top right). This mask is segmented to create a set of regions of interest (ROIs) around each cell (bottom left). Integral density is measured in each ROI and background is subtracted, as described in the Methods section of the Paper, to generate the time trajectory of Ca^{2+} in the cytoplasm (bottom right).

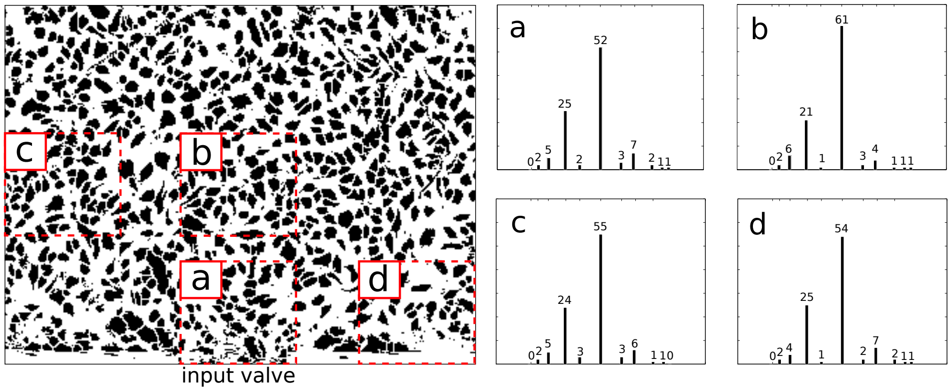

Figure D: Heterogeneity control for CIAD imaging. We measured skipping pattern histograms under pulse stimulation, as shown on the right, in the regions of the CIAD shown on the left. No significant qualitative differences are found between them.

spikefilter

- input = Zspike (spike heights)
- output = binary_sequence

```
for  k = 1:length(Zspike)

    % Define local spikes: four closest neighbours
    mina = max(k-2,1)
    maxa = min(k+2,length(Zspike))
    ind = mina:maxa %index of local spikes

    % Calculate mean and standard deviation of local
    meanlocal = mean(Zspike(ind))
    stdlocal = std(Zspike(ind))

    % Accept or reject spikes: binary sequence
    if  Zspike(k) < meanlocal &  stdlocal > 0.4*meanlocal

        binary_sequence(k)=0

    else

        binary_sequence(k)=1

    end if

end for
```

patternsearch

- input: binary_sequence
- output = list of patterns

```
Npatterns=0

% look for [1,0] "promoters"
for i = 1:length(binary_sequence)-1
    win = [ binary_sequence(i),binary_sequence(i+1) ]

    if  win == [1,0]
        k = k+1
        promoter(k) = i %promoter possitions
    end if

end for

% Analyze patterns within promoters
maxlength=6 %maximum pattern length allowed
for  i=1:length(promoter)-1

    index = promoter(i)+1:promoter(i+1)
    sub_sequence = binary_sequence(index)

    Nones=0
    if  length(sub_sequence) <= maxlength

        for  j = 1:length(sub_sequence)

            %count ones
            if  sub_sequence(j) == 1
                Nones = Nones+1
            end if

        end for

    end if

    Npatterns = Npatterns+1
    pattern(Npatterns) = Nones/length(sub_sequence)

end for
```

Figure E: Pseudo-code of the spike filtering (left) and pattern search (right) algorithms.

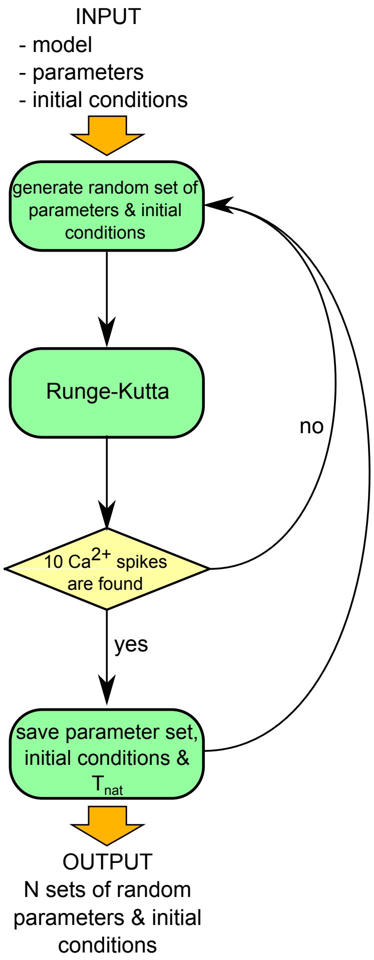


```
Nsets = 0
while ( Nsets < 20,000 )

  % Random sets
  % Generate random set parameters around original ones
  for  i=1:Nparam
     generate random number R1 %uniformly dist. [0,1]
     rand_param(i) = original_param(i)*R1*10
  end for

  % Generate random init. cond. (IC) around original ones
  for  j=1:Nspecies
     generate random number R2 %uniformly dist. [0,1]
     rand_IC(i) = original_IC(j)*R2*10
  end for

  % Solve ODE: cc = calcium, t = time
  [ cc,t ] = runge_kutta(rand_param,rand_IC)

  % Analyze trajectory cc,t: look for spikes
  accepted = false
  Nspikes = 0
  for i=1:length(cc) - 8
        %spike found
        if cc(i+4) > all four points before and after cc(i+4)

           Nspikes=Nspikes+1
           Tspike(Nspikes) = t(i+4)

           %conditions accepted: 10 spikes found
           if Nspikes == 10
              accepted=true
              Nsets = Nsets + 1
           end if

        end if

  end for

  % save results
  if accepted == true
     Tnat = mean( diff(Tspikes) ) %average spiking period
     save rand_IC, rand_param, Tnat
  end if

end while
```

Figure F: Flow chart (left) and pseudo-code (right) for choosing random ICs and PVs and determining the natural period. The pseudo-code illustrates uniform sampling.

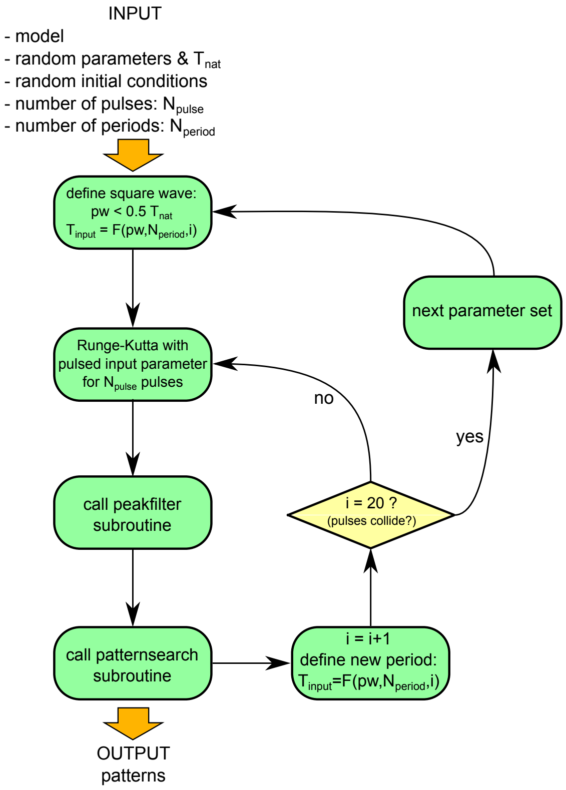


```
for  k = 1:20,000

    % load condtions
    read rand_param,rand_IC,Tnat

    % define pulse width
    pw=(1/8)*Tnat

    % loop over 19 different periods
    for  i = 1:19

        Tinput = pw*20/i

        % Solve pulsed ODE: returns spikes heights and positions
        [ Zspike, Tspike ] = runge_kuttaPULSE(rand_param,rand_IC,Tinput)

       % Filter peaks: binary sequence
       binary_sequence = peakfilter(Zspike)

       % Find patterns
       patterns = patternsearch(binary_sequence)

       save patterns

    end for

end for
```

Figure G: Flow chart (left) and pseudo-code (right) for pattern counting.

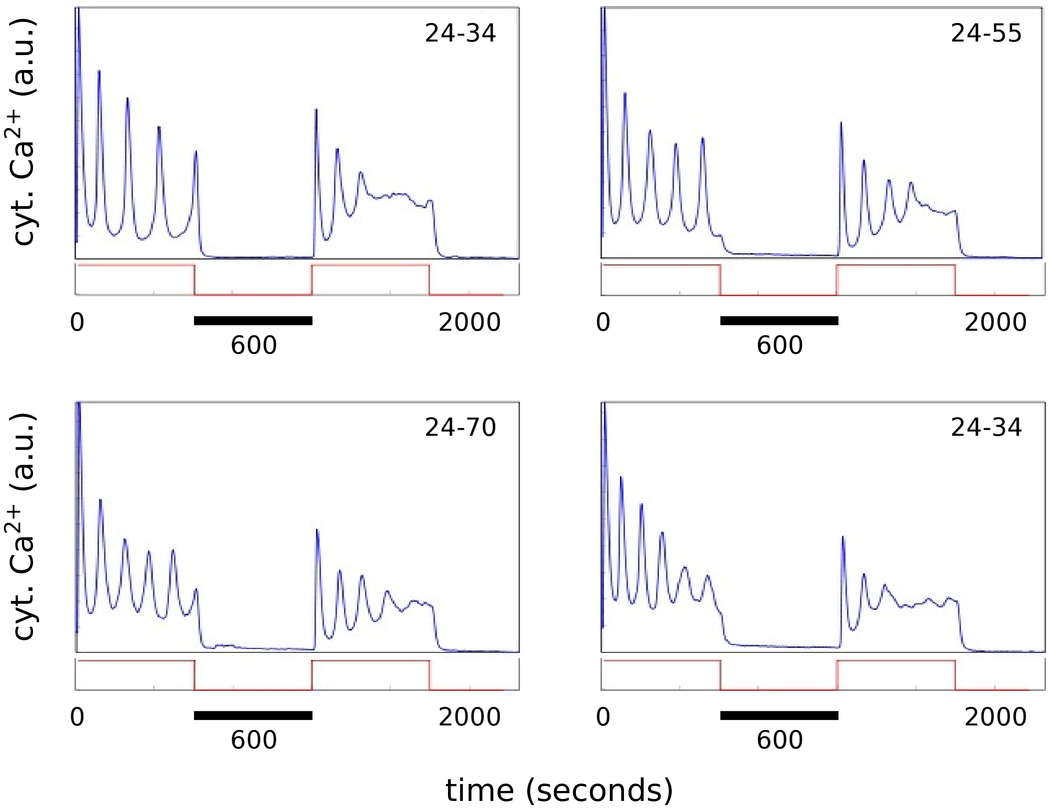

Figure H: Absence of photobleaching. Cells were subjected to two steps of histamine with a gap of 10 minutes between the steps. The response to the second step was similar to that of the first, suggesting that photobleaching was not the dominant reason for the decrease in spike amplitude.

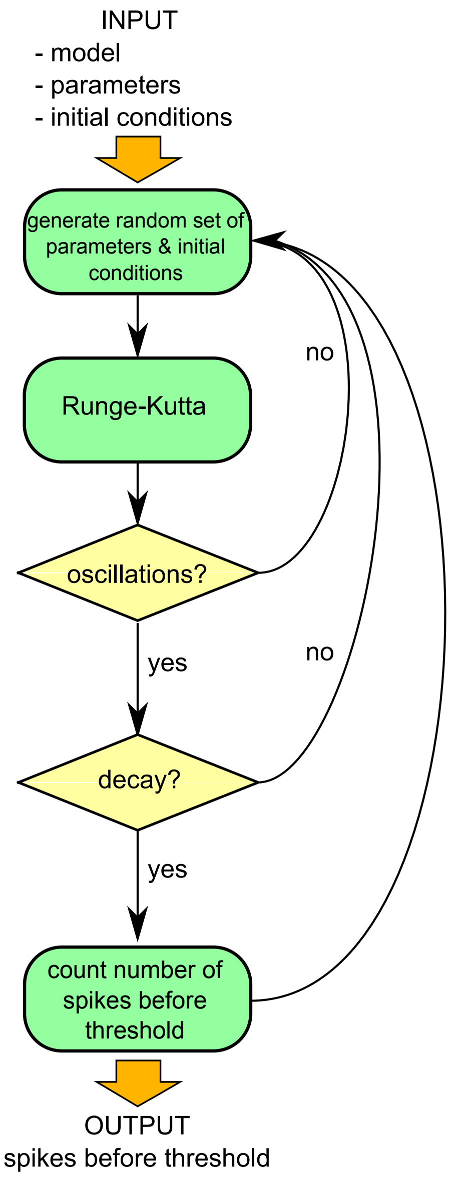


```
Nsets = 0
while ( Nsets < 200,000

  % Random sets
  % Generate random set parameters around original ones
  for  i=1:Nparam
     generate random number R1 %uniformly dist. [0,1]
     rand_param(i) = original_param(i)*R1*10
  end for

  % Generate random init. cond. (IC) around original ones
  for  j=1:Nspecies
     generate random number R2 %uniformly dist. [0,1]
     rand_IC(i) = original_IC(j)*R2*10
  end for

  % Solve ODE: cc = calcium, t = time
  [ cc,t ] = runge_kutta(rand_param,rand_IC)

  % Check if the system oscillates
  oscillates = false
  if  10 spikes
     oscillates = true
  end if

  % Analyze trajectory cc,t: look for decay
  accepted = true
  k = find(max(cc))  %locates the index of the highest spike
  % Analyze trajectory starting at the highest spike
  for  j= k+1:length(cc)
     if cc(j) > cc(j-1)
        accepted = false
        exit
     end if
  end for

  % Count spikes before threshold (SBT)
  if accepted = true
        SBT = 0
        j= 0
        threshold = 0.25*(max(cc) - min(cc)) + min(cc)
        while  cc(j) > threshold
           j=j+1
           SBT = SBT+1
        end while
        save SBT
  end if

end while
```

Figure I: Flow chart (left) and pseudo-code (right) for nonlinear amplitude analysis. The pseudo-code illustrates uniform sampling.

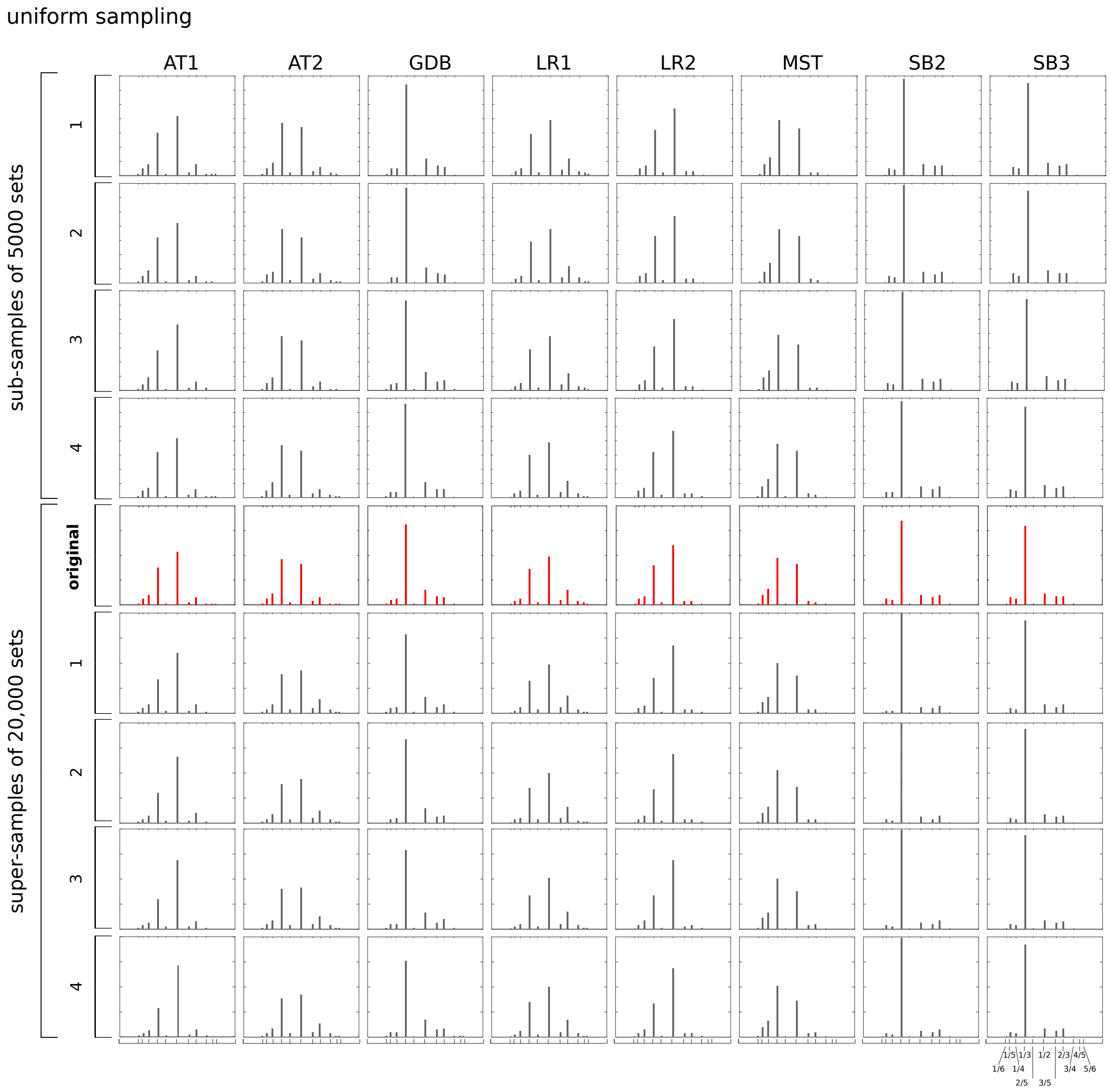

Figure J: Effect of sample size on nonlinear frequency analysis under uniform sampling. Each column corresponds to the model annotated at the top. Each row corresponds to a sample of sets of ICs and PVs. The row in the middle, coloured in red, corresponds to the sample of 20,000 sets used in Paper Figure 5. The four rows above the middle were obtained by random sub-sampling of 5000 sets from among those 20,000 sets. The four rows below the middle were obtained by independently generating further samples of 20,000 sets.

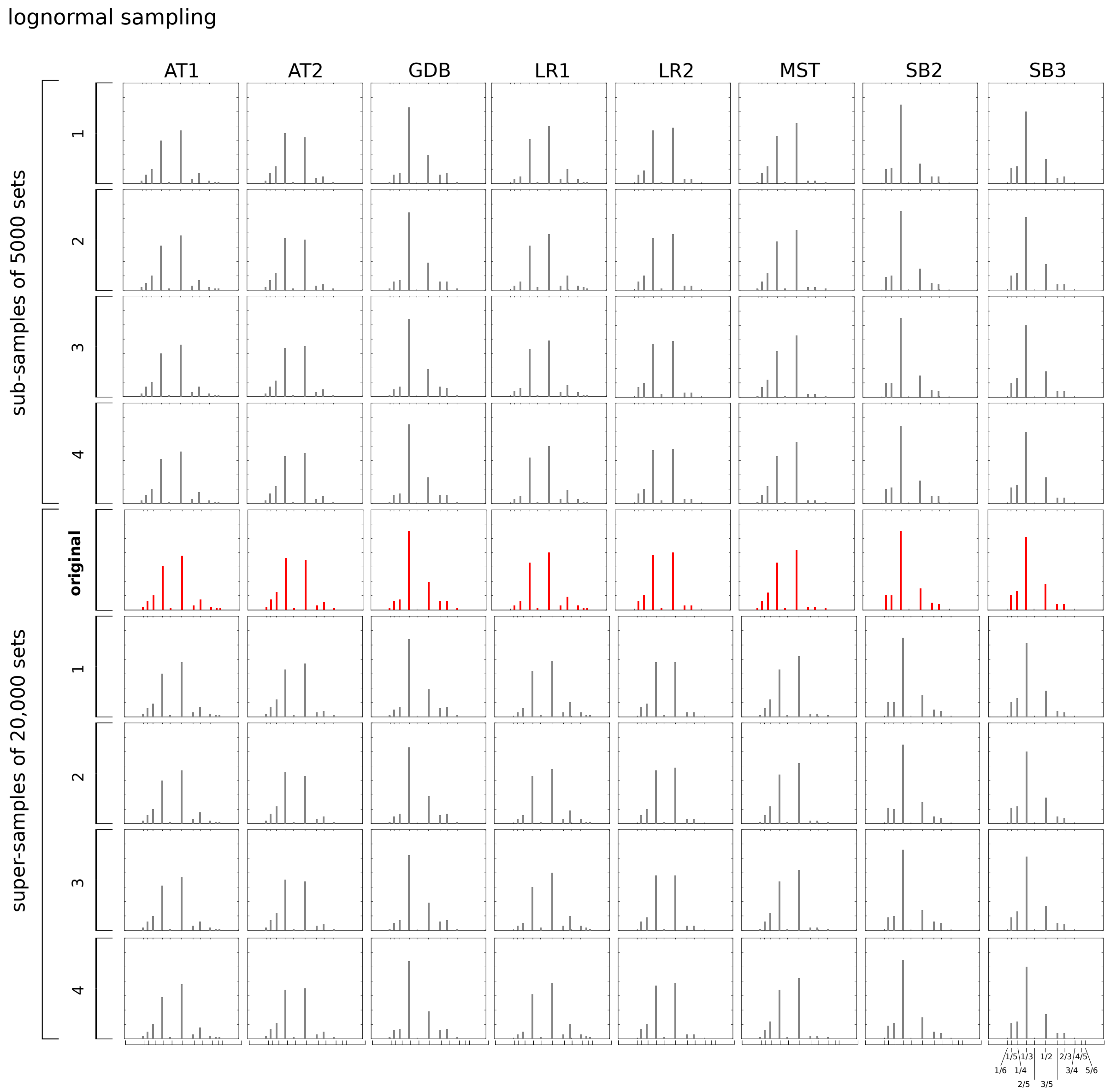

Figure K: Effect of sample size on nonlinear frequency analysis under lognormal sampling, following the same procedure and layout as in Figure J.

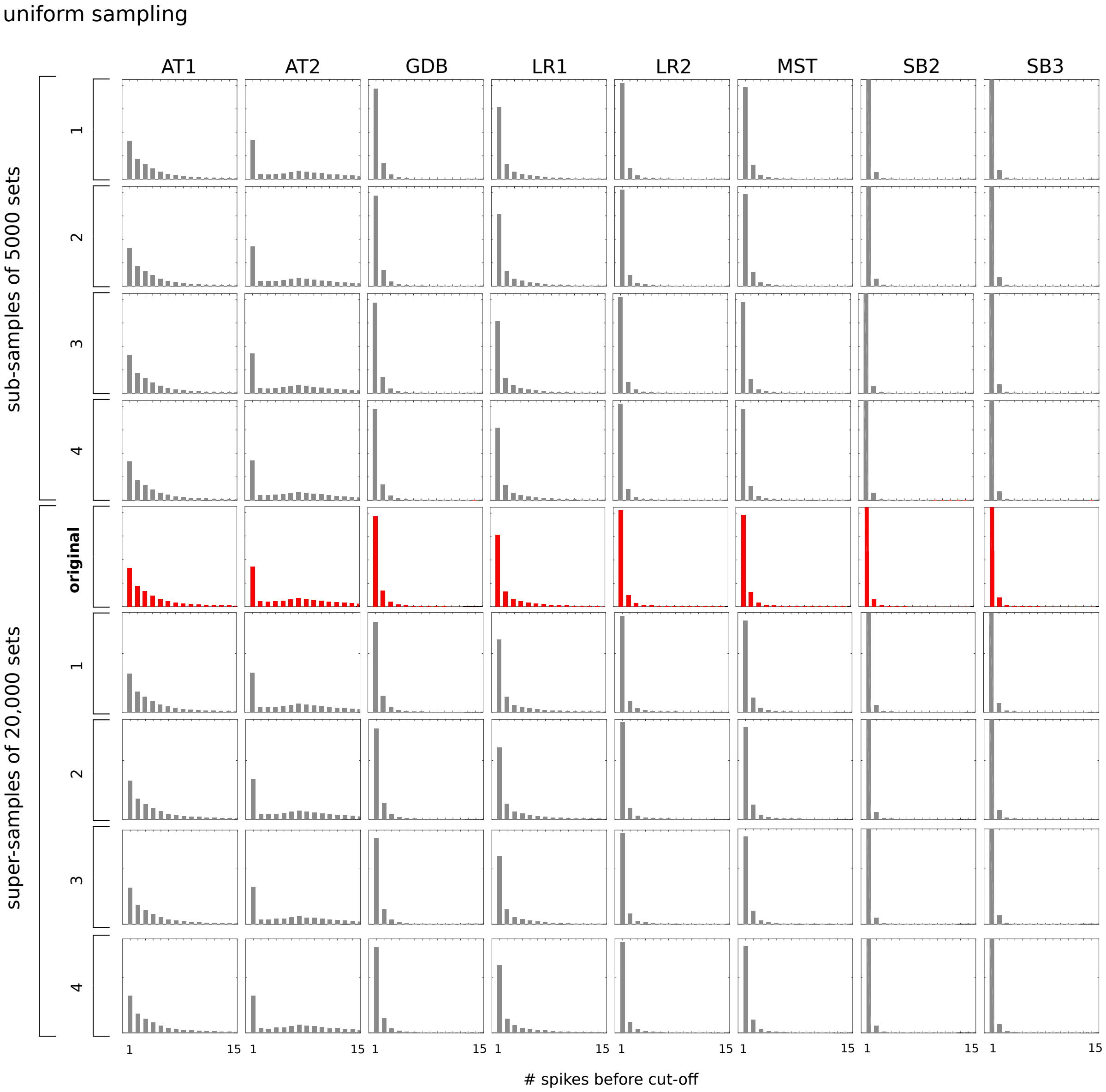

Figure L: Effect of sample size on nonlinear amplitude analysis under uniform sampling, following the same procedure and layout as in Figure J.

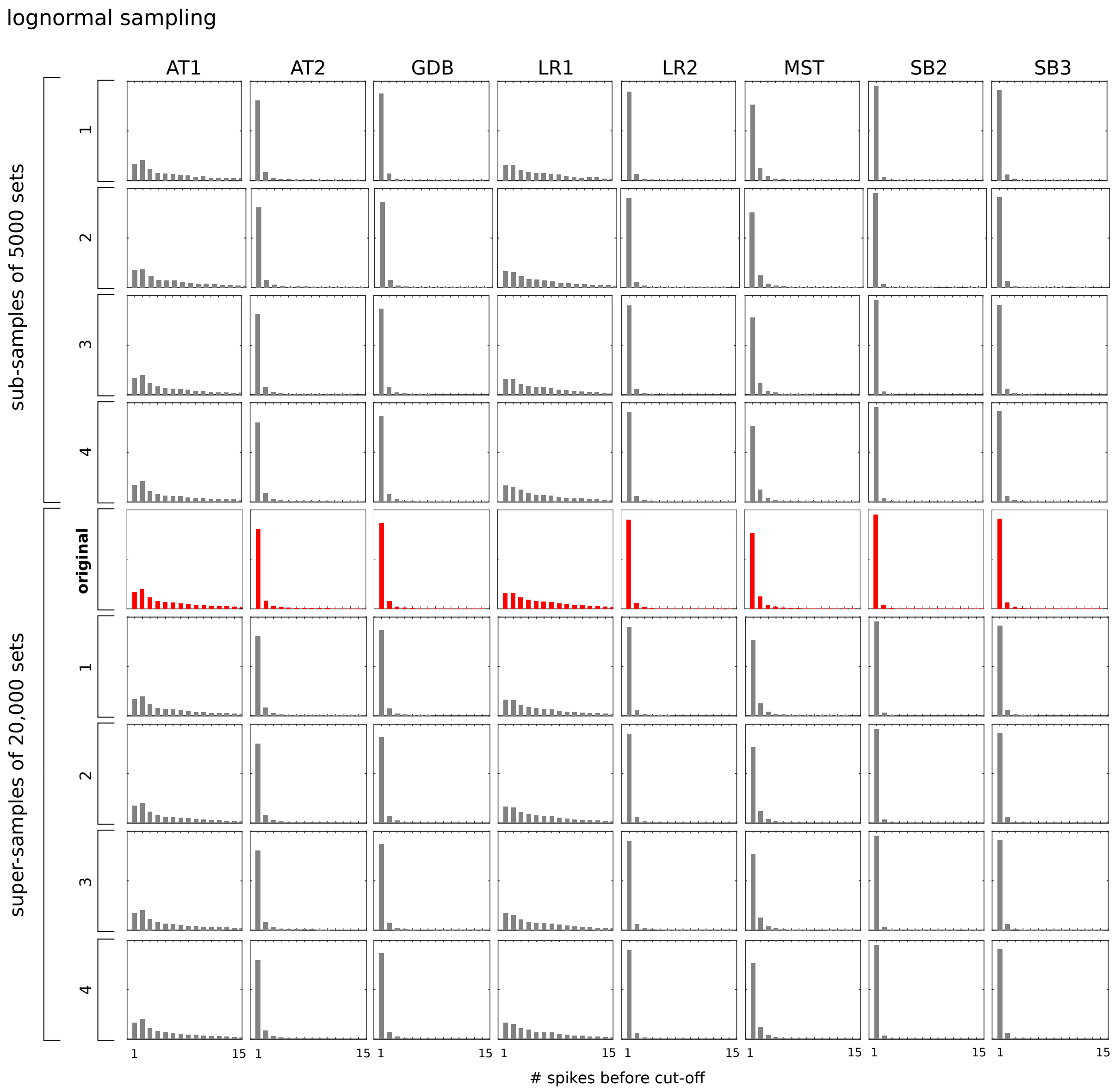

Figure M: Effect of sample size on nonlinear amplitude analysis under lognormal sampling, following the same procedure and layout as in Figure J.
